# Technology-Enhanced Strategies to Optimize Positive End-Expiratory Pressure in Patients Receiving Invasive Mechanical Ventilation: A Systematic Review and Meta-Analysis

**DOI:** 10.1097/CCM.0000000000007144

**Published:** 2026-05-13

**Authors:** Adam J. Boulton, Adel Elfeky, Rachel Court, Amy Grove, Anna Wilson, Peter Auguste, Daniel Clayton, Daniel Gallacher, Ewan C. Goligher, Catherine MacLeod Hall, Daniel F. McAuley, Gavin D. Perkins, Barnaby R. Scholefield, Marion Thompson, Joyce Yeung, Yen-Fu Chen, Keith Couper

**Affiliations:** 1 Warwick Clinical Trials Units, Warwick Medical School, University of Warwick, Coventry, United Kingdom.; 2 Warwick Evidence, Warwick Medical School, University of Warwick, Coventry, United Kingdom.; 3 Centre for Evidence and Implementation Science (CEIS), School of Social Policy and Society, University of Birmingham, Birmingham, United Kingdom.; 4 Critical Care Unit, University Hospitals Birmingham NHS Foundation Trust, Birmingham, United Kingdom.; 5 Interdepartmental Division of Critical Care Medicine, University of Toronto, Toronto, ON, Canada.; 6 Wellcome-Wolfson Institute for Experimental Medicine, School of Medicine, Dentistry and Biomedical Science, Queen’s University Belfast, Belfast, Northern Ireland, United Kingdom.; 7 Department of Critical Care, Royal Victoria Hospital, Belfast, Northern Ireland, United Kingdom.; 8 Department of Critical Care Medicine, Hospital for Sick Children, Toronto, Canada.; 9 Patient and Public Contributor, Warwick Medical School, University of Warwick, Coventry, United Kingdom.

**Keywords:** critical care, individualized medicine, positive end-expiratory pressure, positive-pressure respiration intensive care, respiratory distress syndrome

## Abstract

**Objectives:**

To undertake a systematic review evaluating the clinical and cost-effectiveness of technology-enhanced positive end-expiratory pressure (PEEP) optimization strategies in adults and children receiving invasive mechanical ventilation on an ICU.

**Data Sources:**

We searched key electronic databases (including MEDLINE and Embase) from inception to July 2024.

**Study Selection:**

We included randomized studies examining clinical or cost-effectiveness of technology-enhanced PEEP optimization strategies compared with standard care or an alternative PEEP optimization strategy in adults and children. The primary outcome was duration of mechanical ventilation and secondary outcomes were clinical effectiveness (e.g., mortality) and efficacy (e.g., PEEP).

**Data Extraction:**

Two reviewers independently assessed eligibility, extracted data, assessed risk of bias (Revised Cochrane tool) and performed Grading of Recommendations Assessment, Development and Evaluation evidence certainty assessments.

**Data Synthesis:**

Our database and trial register search retrieved 8845 results, of which 34 studies (2951 patients) were included. Eight studies were at low risk of bias. Across studies, 7 technologies were evaluated, most commonly esophageal balloon measurement of transpulmonary pressure (10 studies), electrical impedance tomography (7 studies), pressure-volume curve analysis (6 studies), and fully automated closed-loop ventilation (5 studies). Meta-analysis used random-effects models. Duration of mechanical ventilation was reported in only three studies (172 patients, two technologies) and there was no effect compared with standard care (mean difference –0.06 d; 95% CI, –0.20 to 0.09; very low-certainty evidence).

For 28-day mortality (10 studies; 1,719 patients; six technologies), technology-enhanced PEEP optimization reduced 28-day mortality (risk ratio 0.69; 95% CI, 0.52–0.93; very low-certainty evidence). No significant differences were found for other clinical-effectiveness outcomes. We identified no evidence in children or on cost-effectiveness.

**Conclusions:**

Technology-enhanced PEEP optimization strategies did not reduce duration of mechanical ventilation, but these technologies may reduce mortality. Evidence certainty was low or very low, highlighting the urgent need for adequately powered randomized trials.

**Registration:**

PROSPERO (CRD42024555390).

**KEY POINTS**

**Question:** Does technology-enhanced positive end-expiratory pressure (PEEP) optimization improve clinical or cost-effectiveness outcomes in mechanically ventilated adults and children compared with standard care or alternative strategies?

**Findings:** In this systematic review and meta-analysis of 34 randomized trials (2951 patients), technology-enhanced PEEP optimization did not significantly reduce the duration of mechanical ventilation (3 studies; 172 patients), but may reduce 28-day mortality (10 studies; 1719 patients). Both findings are based on very low-certainty evidence.

**Meaning:** Technology-enhanced PEEP optimization did not reduce the duration of mechanical ventilation but may improve survival. High-quality trials are urgently needed to robustly assess clinical and cost-effectiveness.

Each year, between 13 and 20 million patients receive invasive mechanical ventilation across the world (1). Mechanical ventilation is life-saving, but it can also be injurious. The way in which mechanical ventilation is delivered can minimize lung injury, alter respiratory physiology, and influence patient outcomes (2–5).

Positive end-expiratory pressure (PEEP) is a fundamental component of invasive mechanical ventilation. Optimal PEEP supports lung recruitment and improves oxygenation (6–8). However, inappropriate PEEP can cause lung injury, including by overdistention and the associated baro- and volu-trauma (9, 10). There is a complex dynamic interplay between PEEP and other ventilatory pressures, as well as distant effects on abdominal, cardiovascular, intracranial, and renal physiology (11–13).

The optimal method for selecting and titrating PEEP is unknown (14). Guideline recommendations are limited to supporting sufficient or high-PEEP strategies in certain patient groups (15–17). For the bedside clinician, the concept of a physiologically guided personalized strategy is attractive, however reliably evaluating patients is challenging and misclassification can introduce harm (18, 19). Innovative technologies may help guide personalized strategies and improve outcomes (20, 21). A technology-based approach could offer adaptive strategies that are agile to changing respiratory mechanics. Our recent scoping review identified an emerging evidence base for technology-enhanced PEEP optimization (22). Given that recent reviews have typically focused on a single technology (23), we identified the need to undertake a systematic review to evaluate the clinical and cost-effectiveness of different technology-enhanced PEEP optimization strategies in adults and children receiving invasive mechanical ventilation on an ICU.

## METHODS

The protocol was prospectively registered on the PROSPERO (CRD42024555390).

This article is written in line with the Preferred Reporting Items for Systematic Reviews and Meta-Analyses statement guidelines (**Supplementary material**, https://links.lww.com/CCM/H961) (24).

### Eligibility Criteria and Outcomes

We defined our eligibility criteria and outcomes using the Population, Intervention, Comparator, Outcome framework.

#### Population

Adults (18-yr and older) or children (1-mo to 17-yr old) receiving invasive mechanical ventilation on an ICU. Newborns aged younger than 1-month and individuals receiving mechanical ventilation outside of an ICU (e.g., operating theater) were excluded.

#### Intervention

Technology-enhanced PEEP optimization strategies, which we defined as a strategy that used information from a novel technology (beyond those used in standard care) to inform the initial selection and/or subsequent titration of PEEP. Examples of these technologies include electrical impedance tomography, esophageal balloon measurement of transpulmonary pressure, pressure–volume curve analysis, and ultrasonography. This also included fully automated closed-loop ventilation modes that automatically titrate PEEP, namely INTELLiVENT-Adaptive Support Ventilation (ASV) mode.

#### Comparator

Standard care or an alternative PEEP optimization strategy. Typically, standard care would comprise determining PEEP using ventilatory pressures or blood gas values (e.g., based on ARDSnet tables).

#### Outcomes

Our primary outcome was duration of mechanical ventilation. Other clinical outcomes were mortality (ICU/hospital at 28-d or 30-d combined), ventilator-free days (combined 28/30-d), ICU length of stay, hospital length of stay, health-related quality of life, ventilatory parameters (e.g., PEEP/plateau pressure/driving pressure/mechanical power), and clinically important adverse events. Pressures were measured in centimeters of water (cm H_2_O) and mechanical power was measured in Joules per minute.

Health-economic outcomes included costs, quality-adjusted life years, and incremental cost-effectiveness ratios.

For clinical and efficacy outcomes, we included only randomized studies, including parallel-group randomized controlled trials (RCTs) and randomized crossover trials. For health-economic outcomes, we included full economic evaluations (cost-benefit, cost-effectiveness, cost-utility, cost-consequence analyses, cost-minimization studies) and cost studies. Grey literature, including studies published only as abstracts and research letters, was eligible for inclusion.

We did not impose any limits on language or year of publication.

### Information Sources and Search Strategy

Our information specialist iteratively developed a search strategy with input from a clinical subject expert. The following databases were searched from inception to July 2024: MEDLINE (Ovid), Embase (Ovid), Cochrane Library, Science Citation Index and Conference Proceedings Citation Index—Science (Web of Science), CEA Registry, Dissertations & Theses Global database (ProQuest). No date or language limits were applied. Searches were based on terms for optimization (or technologies/methods used for optimization) and PEEP, using both free text and controlled vocabulary terms. Relevant study type filters in the larger medical and science databases were used (RCTs, systematic reviews, economic evaluations) (25–27). Full details of the searches are available in the Supplementary materials (https://links.lww.com/CCM/H961). Records were imported to EndNote software and systematically de-duplicated using a process informed by the University of Leeds method (28). Grey literature searches were also undertaken of: dissertations and theses, trial registers, relevant manufacturer websites, reference lists of included studies and recent, relevant reviews, discussion with collaborator subject experts, and a trial register search (Supplementary material, https://links.lww.com/CCM/H961).

### Study Selection Process and Data Extraction

Titles and abstracts for all returned articles were reviewed independently by at least two authors. Full texts were obtained for articles of interest and these were independently reviewed by at least two authors against the eligibility criteria, with any discrepancies resolved by discussion with a third adjudicating author. Reviewers were academics and critical care clinicians. Data were extracted into a data extraction form and independently checked by a second author (data items detailed in Supplementary material, https://links.lww.com/CCM/H961). We also recorded the reporting of key demographic information in line with the PROGRESS-PLUS (place of residence; race, ethnicity, culture, language; occupation; gender, sex; religion; education; socioeconomic status; social capital-plus) criteria (29, 30).

### Risk of Bias and Certainty of Evidence Assessment

Individual study risk of bias was assessed independently by two authors, with involvement of a third adjudicating author where required. The Revised Cochrane risk of bias tool (RoB 2) was used to assess risk of bias, with the crossover trial version used where appropriate (31). Risk of bias plots were produced using the *robvis* R package (R Foundation for Statistical Computing, Vienna, Austria) (32). The Grading of Recommendations Assessment, Development and Evaluation (GRADE) approach was used to assess the certainty of evidence for each outcome and technology and facilitated by GRADEPro software (Supplementary material, https://links.lww.com/CCM/H961) (33, 34).

### Evidence Synthesis Strategy

The strategy for evidence synthesis was guided by the Cochrane Handbook for Systematic Reviews of Interventions (35). We initially tabulated studies to describe key characteristics. We grouped studies by the technology used for PEEP optimization, rather than the physiologic measurement used for PEEP optimization. For duration of mechanical ventilation, we preferentially extracted data for survivors for the primary analysis. For studies comparing a PEEP optimization strategy with standard care, we synthesized study findings in a meta-analysis using a random-effects model. The findings of studies that compare two PEEP optimization strategies are described in a narrative synthesis. We planned three subgroup analyses, namely type of PEEP optimization strategy, adults compared with children, and presence of acute respiratory distress syndrome (ARDS) at randomization. In each forest plot, we separated studies by technology and provide an estimate of effect for the specific technology and an overall estimate of the effect of technology-enhanced PEEP optimization for that outcome. We also undertook post hoc sensitivity analyses for the outcomes of mechanical ventilation duration and mortality in which we grouped studies by measured physiologic parameter and sequential exclusion of technology groups from meta-analyses.

The findings of meta-analyses are presented as risk ratio and 95% CIs for dichotomous outcomes and as mean differences and 95% CIs for continuous outcomes. We additionally calculated the absolute difference for dichotomous outcomes to inform GRADE certainty assessments. The *I*^2^ statistic was calculated to quantify the proportion of total variance attributed to statistical heterogeneity between studies. All analyses were conducted using STATA, Version 18 (StataCorp, College Station, TX).

## RESULTS

**Figure 1.**
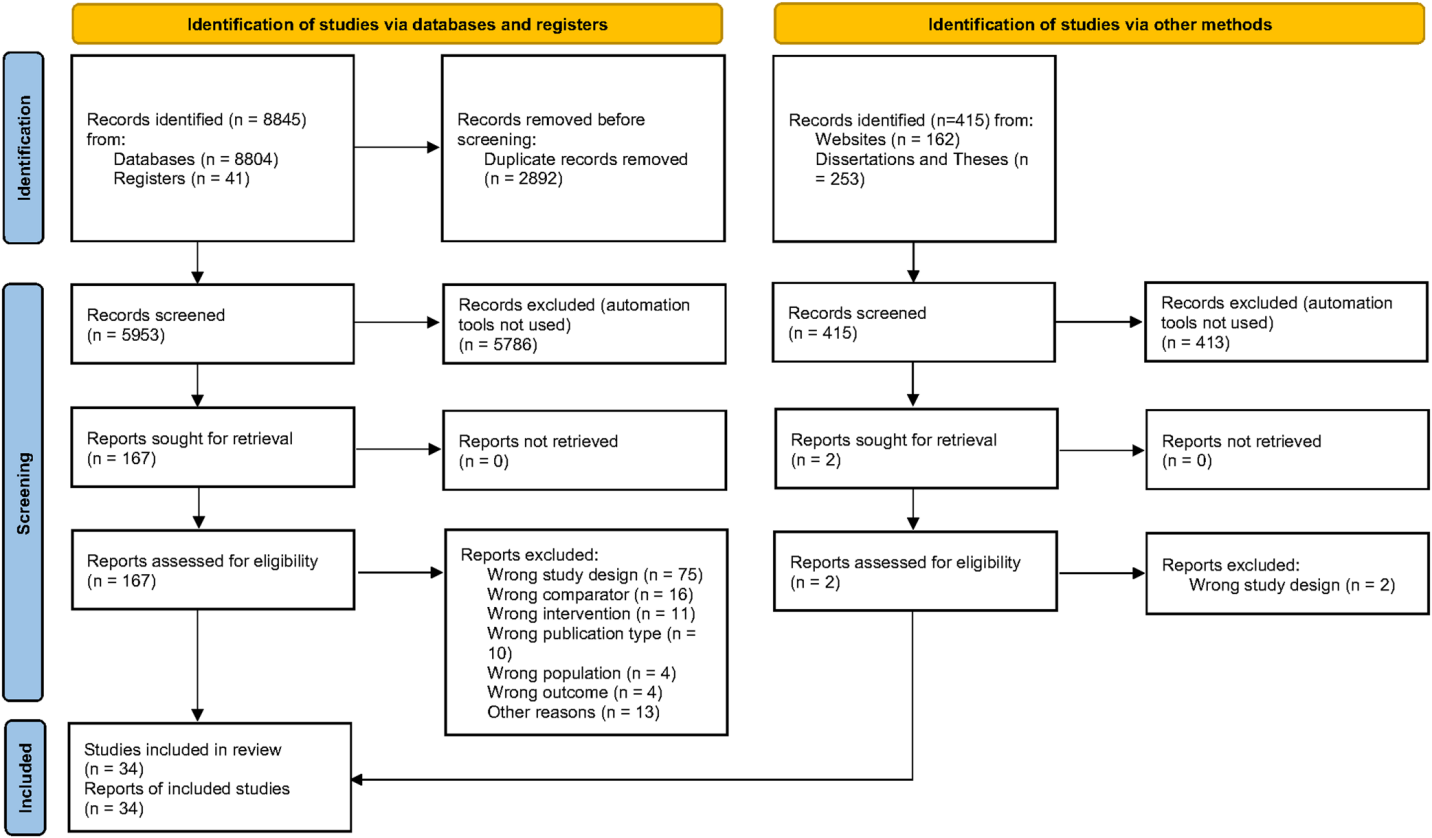
Preferred Reporting Items for Systematic Reviews and Meta-Analyses diagram.

Our database and trial register search identified 8845 results. After de-duplication, we screened 5953 citations and 167 underwent full-text screening, with 34 studies meeting eligibility criteria (36–69). No further studies were identified from grey literature searching (**Fig. 1**).

### Overview of Included Studies

The 34 included studies reported data on a total of 2951 participants. Twenty-two of the included studies used parallel-group design and 12 used a randomized crossover design (**Supplementary Tables S1** and **S2**, https://links.lww.com/CCM/H961). All studies included only adults. Most were undertaken in Asia (*n* = 14) and Europe (*n* = 12), with the remainder in North America (*n* = 4), South America (*n* = 2), and Africa (*n* = 1). One study recruited participants across South America and Europe. Seventeen studies were published since 2020. We identified no cost-effectiveness studies.

Across included studies, seven technology-enhanced PEEP optimization strategies were identified. Ten studies used esophageal balloon measurement of transpulmonary pressure (39, 40, 45, 53, 57, 63, 65–68), seven studies used electrical impedance tomography (37, 44, 47, 48, 51, 55, 62), six studies used pressure–volume curve analysis (36, 48–50, 54, 56), five studies used fully automated closed-loop ventilation (INTELLiVENT-ASV) (38, 41, 43, 52, 69), four studies used static respiratory compliance measurement (42, 57–59), and two studies each used nitrogen wash-in/washout measurement of functional residual capacity (46, 60) and lung ultrasound (61, 64). Four studies compared a technology-enhanced PEEP optimization strategy with another technology-enhanced PEEP optimization strategy (45, 48, 57, 62), whereas the remainder compared with standard care. Comparisons between technology-enhanced strategies were electrical impedance tomography vs. pressure–volume curve analysis (48), electrical impedance tomography vs. esophageal balloon (62), esophageal balloon vs. static respiratory compliance (57), and one study compared two different approaches to esophageal balloon measurement of transpulmonary pressure (at end inspiration vs. at end expiration) (45). Based on categorization by measured physiologic parameter, 19 studies concerned respiratory mechanics, 10 regional ventilation and aeration, 5 gas exchange and control, and 2 lung volume (**Supplementary table S3**, https://links.lww.com/CCM/H961).

### Risk of Bias

The risk of bias assessments are visualized in **Figures S1** and **S2** (https://links.lww.com/CCM/H961). Among the 22 parallel-group RCTs, 8 were at low risk of bias, 10 had some concerns, 4 were at high risk of bias. Those at high risk of bias were due to deviation from intended interventions in three instances and due to missing outcome data in one instance. Among the 12 randomized crossover trials, 9 had some concerns, and 3 were at high risk of bias. Those at high risk of bias were due to deviation from intended interventions in two instances, and due to period and carryover effects in one instance.

### Clinical-Effectiveness Outcomes

**Figure 2.**
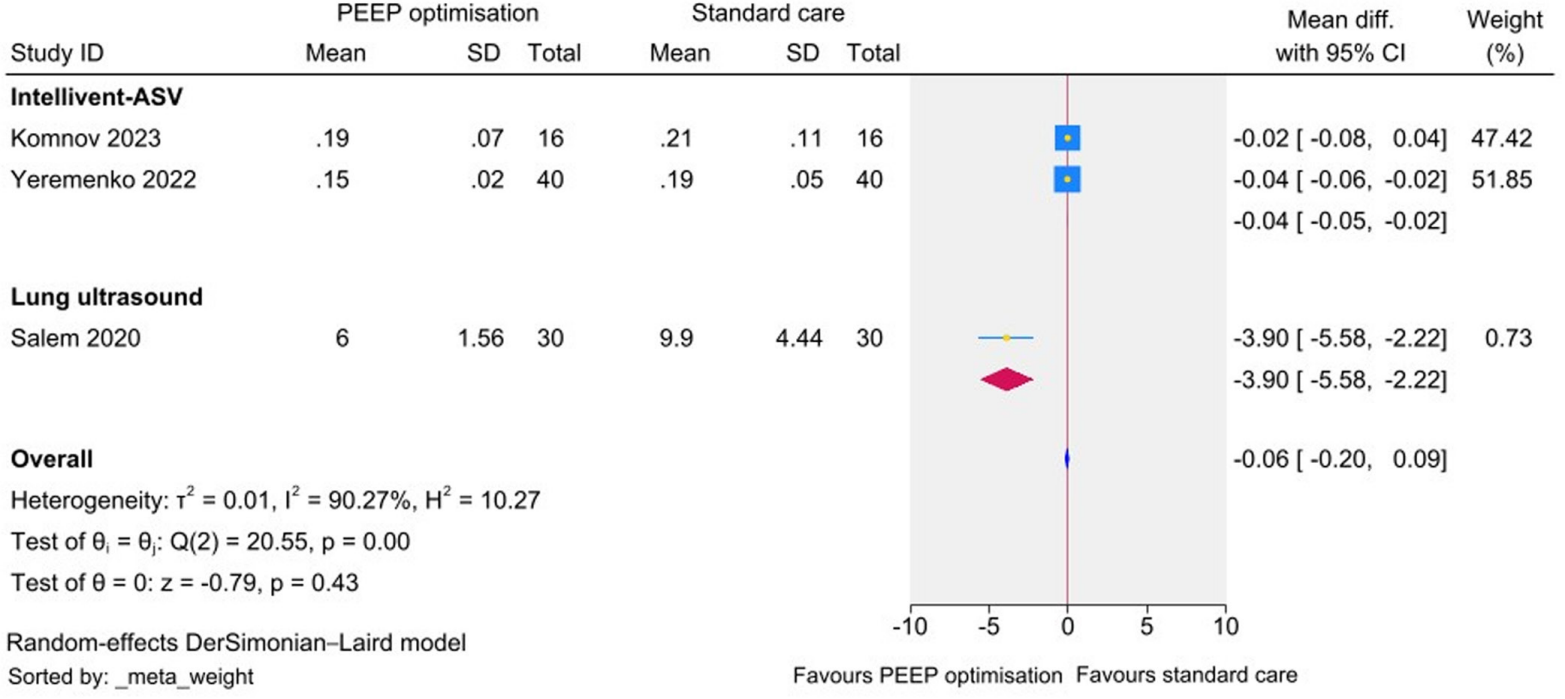
Duration of mechanical ventilation meta-analysis. ASV = Adaptive Support Ventilation, PEEP = positive end-expiratory pressure.

For our primary outcome of duration of mechanical ventilation, we included three studies (two fully automated closed-loop ventilation and one for lung ultrasound) (**Fig. 2**). One trial reported duration in survivors only since no patients died (69); however, for the other two trials it was unclear. Overall, technology-enhanced PEEP-optimization strategies did not reduce duration of mechanical ventilation vs standard care (mean difference –0.06 d; 95% CI, –0.20 to 0.09; three studies; 172 patients; very low-certainty evidence). The observed effect seemed to differ across technologies, with significant favorable effects in the single trial for lung ultrasound, but not in the two trials for fully automated closed-loop ventilation.

**Figure 3.**
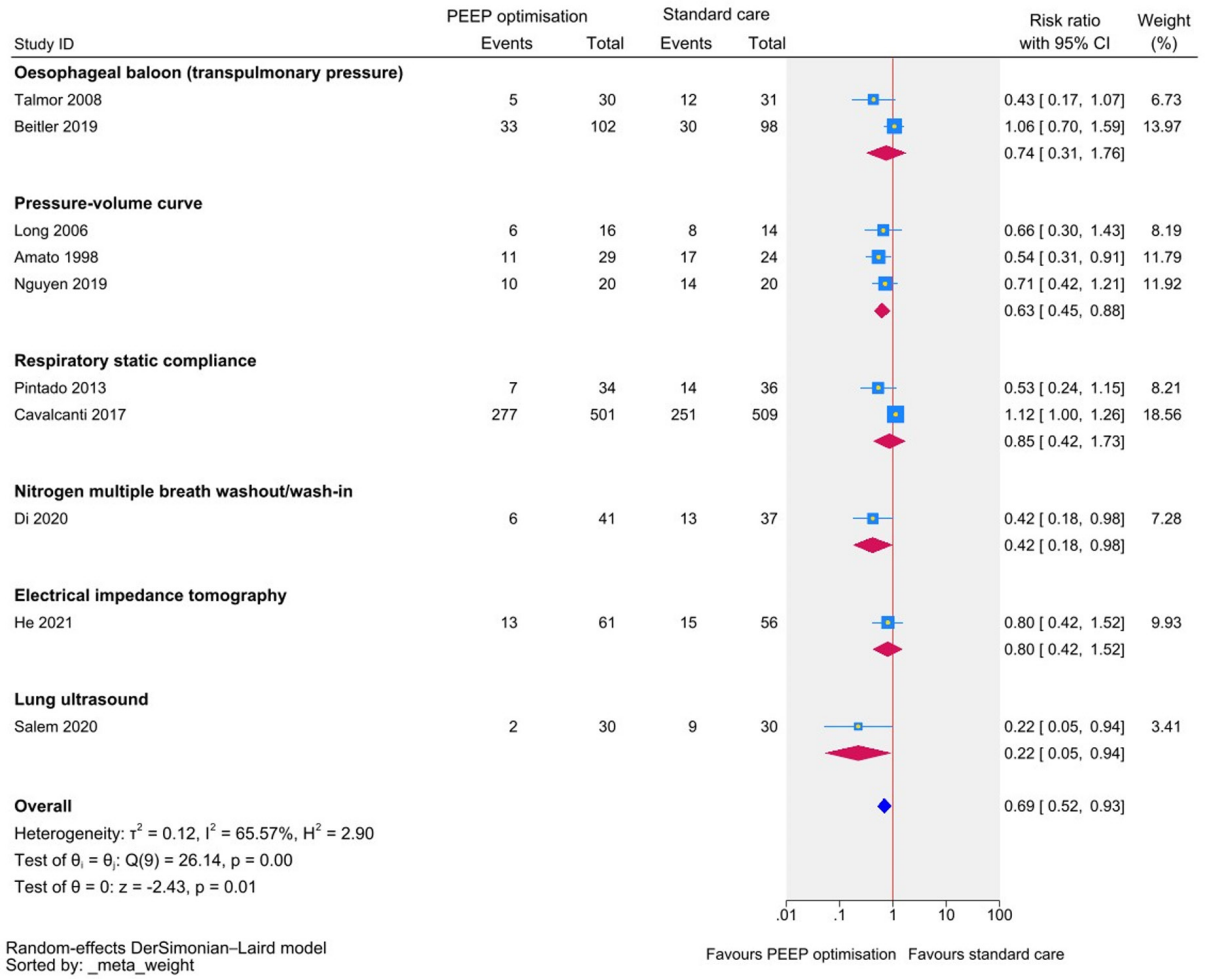
Mortality at 28 days meta-analysis. PEEP = positive end-expiratory pressure.

For 28-day mortality, we included 10 studies (3 pressure–volume curve analysis, 2 esophageal balloon, 2 static respiratory compliance measurement, and 1 each for electrical impedance tomography, lung ultrasound, and nitrogen wash-in/washout) (**Fig. 3**). Across all 10 studies, technology-enhanced PEEP-optimization strategies appeared to reduce 28-day mortality vs. standard care (risk ratio 0.69; 95% CI, 0.52–0.93; 1719 patients; absolute risk reduction 13.9%; 95% CI –21.5% to –3.1%; very low- to low-certainty evidence). The point-estimate for all technologies favored use of technology-enhanced PEEP optimization, but the effect seemed to vary by technology (pressure–volume curve analysis absolute risk reduction 24.9% (95% CI, –37.0 % to –8.1%), lung ultrasound 23.4% (95% CI, –28.5% to –1.8%), nitrogen wash-in/washout 20.4% (95% CI, –20.4% to –0.7%), electrical impedance tomography 5.4% (95% CI, –15.5% to +13.9%), and static respiratory compliance 7.3% (95% CI, –28.2% to +35.5%)). Funnel plot including the 10 studies showed asymmetry (Egger test *p* < 0.001), suggesting possible small study effects (Supplementary material, https://links.lww.com/CCM/H961).

**Figure 4.**
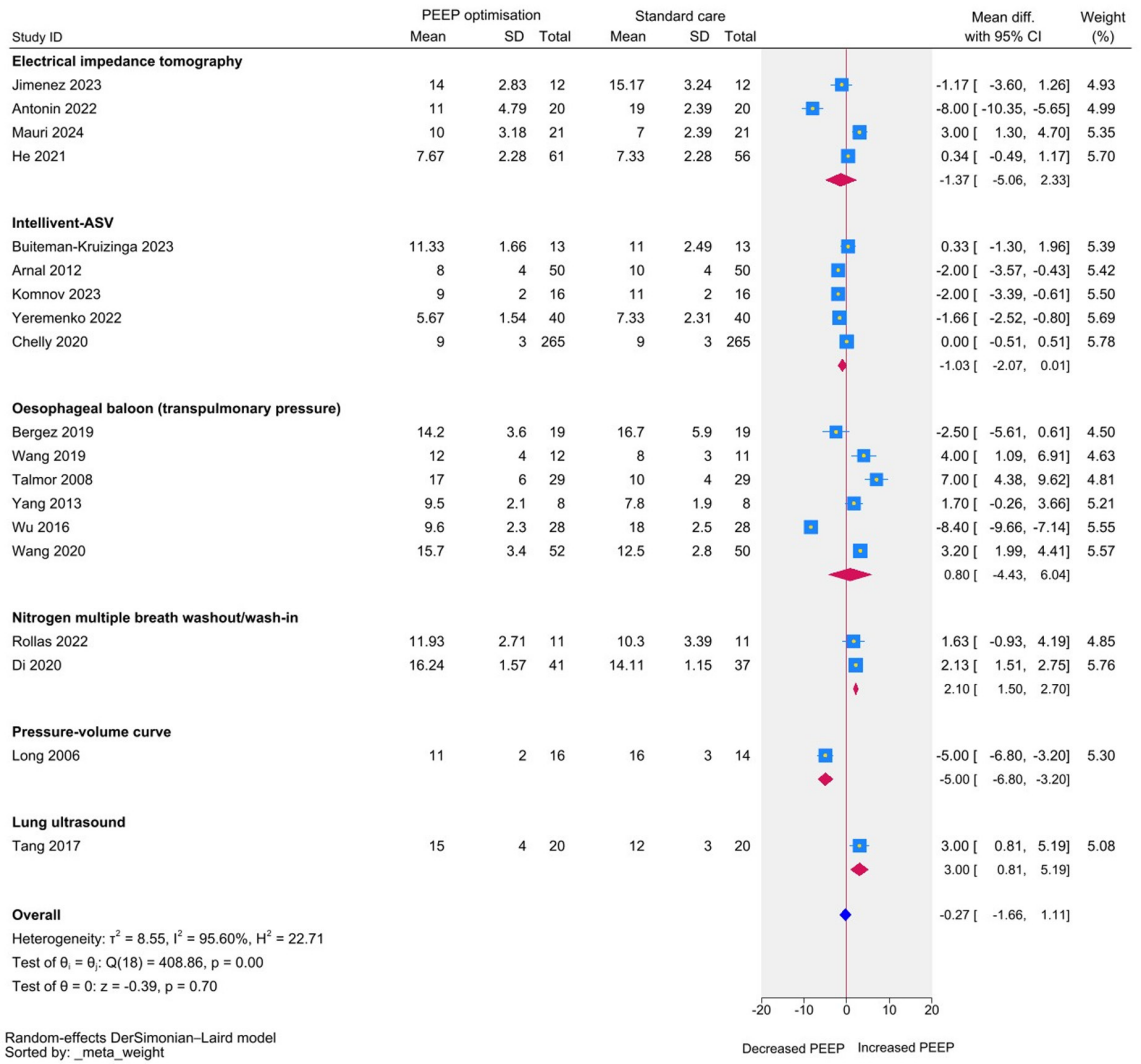
Positive end-expiratory pressure (PEEP) meta-analysis. Mean in cm H_2_O. ASV = Adaptive Support Ventilation.

For ventilator-free days at day 28, we included six studies (two esophageal balloon, two used static respiratory compliance measurement, and one each for electrical impedance tomography, and lung ultrasound) (**Fig. S3**, https://links.lww.com/CCM/H961). Technology-enhanced PEEP optimization strategies did not increase ventilator-free days at day 28 vs standard care (mean difference 0.92 d; 95% CI, –1.97 to 3.81; six studies; 1518 patients; very low- to moderate-certainty evidence). The observed effect seemed to differ across technologies, with significant favorable effects for lung ultrasound, no significant effect for esophageal balloon and electrical impedance tomography, and significantly unfavorable effects for static respiratory compliance measurement.

For ICU length of stay, we included seven studies (three esophageal balloon, two static respiratory compliance measurement, and one each for electrical impedance tomography and lung ultrasound) (**Fig. S4**, https://links.lww.com/CCM/H961). Technology-enhanced PEEP optimization strategies may be associated with an increased ICU length of stay vs standard care (mean difference 1.88 d; 95% CI, 0.22–3.53; seven studies; 1620 patients; very low- to moderate-certainty evidence). The point-estimate for all technologies favored standard care, with the only technology associated with benefit being electrical impedance tomography.

For hospital length of stay, we included five studies (two esophageal balloon, two static respiratory compliance measurement, and one for fully automated closed-loop ventilation) (**Fig. S5**, https://links.lww.com/CCM/H961). Technology-enhanced PEEP optimization strategies did not reduce hospital length of stay vs. standard care (mean difference 4.91 d; 95% CI, –5.83 to 15.66; five studies; 1462 patients; very low-certainty evidence).

Two of the four studies comparing technology-enhanced PEEP optimization strategies reported clinical-effectiveness outcomes (48, 57). Compared with static compliance, esophageal balloon significantly reduced the duration of mechanical ventilation (mean 6.3 d vs. 15.1 d; 17 patients; *p* = 0.012). Compared with pressure–volume curve analysis, electrical impedance tomography significantly reduced survival to hospital discharge (69.0% vs. 44.4%; 87 patients; *p* = 0.02), whereas duration of mechanical ventilation and ICU length of stay were not significantly different.

No study reported health-related quality of life outcomes.

### Safety and Efficacy Outcomes

Technology-enhanced PEEP optimization strategies appeared to increase the risk of pneumothorax (risk ratio 2.28; 95% CI, 1.02–5.09; three studies; 1,248 patients; very low- to low-certainty evidence) but not barotrauma, using individual study definitions of pneumomediastinum, pneumothorax, or subcutaneous emphysema (risk ratio 1.38; 95% CI, 0.71–2.67; six studies; 1518 patients; very low-certainty evidence) (**Figs. S6** and **S7**, https://links.lww.com/CCM/H961).

Technology-enhanced PEEP strategies did not have a statistically significant effect on PEEP (mean difference –0.27 cm H_2_O; 95% CI, –1.66 to 1.11; 19 studies; 1454 patients; very low- to low-certainty evidence) (**Fig. 4**) or plateau pressure (mean difference –2.94 cm H_2_O; 95% CI, –6.14 to 0.27; nine studies; 408 patients; very low-certainty evidence). In contrast, technology-enhanced PEEP optimization strategies reduced both driving pressure (mean difference –1.38 cm H_2_O; 95% CI, –2.23 to –0.53; eight studies; 441 patients; very low- to low-certainty evidence) and mechanical power (mean difference –1.75 J/min; 95% CI, –2.44 to –1.06; six studies; 342 patients; very low-certainty evidence). Full details of the analyses for safety and efficacy outcomes are summarized in **Figures S8-S10** (https://links.lww.com/CCM/H961).

### Subgroup and Sensitivity Analyses

We were unable to undertake our planned subgroup analyses of ARDS at randomization and age group due to the paucity of evidence in patients without ARDS and absence of evidence in children, respectively. Post hoc sensitivity analyses in which we group studies by measured physiologic parameter are available in the Supplementary materials (**Figs. S11** and **S12**, https://links.lww.com/CCM/H961). Our post hoc sensitivity analyses in which we excluded technologies one-by-one for the outcomes of duration of mechanical ventilation and mortality are included in **Supplementary tables S4** and **S5** and **Fig. S13** (https://links.lww.com/CCM/H961). Of note, excluding pressure–volume curve studies from the analysis altered the observed effect for mortality such that it no longer reached statistical significance (risk ratio 0.72; 95% CI, 0.50–1.04).

## DISCUSSION

In this systematic review of 2951 patients across seven technologies and 34 trials, we found that technology-enhanced PEEP optimization did not reduce duration of mechanical ventilation, but that these technologies may reduce mortality. This reduction in mortality should be interpreted with care since there was moderate statistical heterogeneity, as well as funnel plot asymmetry with the two studies of low of bias with the largest samples sizes showing no effect. The effectiveness of technologies may vary. We found no evidence on cost-effectiveness or in children. The certainty of evidence was assessed as low or very low for all outcomes, meaning that the true effect may be substantially different from the estimate, highlighting the need for caution when interpreting these findings.

Our review included seven technology-enhanced PEEP optimization strategies. These range in complexity and cost from technologies that are readily available on many modern-day ventilators, such as pressure-volume curve analysis, to technologies that likely require additional training and equipment, such as electrical impedance tomography. This would present significant implementation challenges for many organization; however, some of these technologies are being used in current clinical practice (70, 71). Although we provide an overall estimate of the clinical-effectiveness of technology-enhanced PEEP optimization strategies, we identified evidence that these technologies may vary in their effectiveness. Our finding of an increased risk in pneumothorax came from only three studies (two esophageal balloon; one respiratory static compliance) but was predominantly driven by the single-respiratory static compliance study. This same study reported fewer ventilator-free days in the intervention group. This warrants careful consideration and is consistent with concerns regarding recruitment maneuvres, highlighting that not all PEEP optimization technologies share the same risk-benefit profiles (15, 16). The process of static compliance measurement may cause harm by barotrauma and increased risk of hemodynamic instability, perhaps in a similar fashion to prolonged recruitment maneuvres (42). The signals of potential harm in pneumothorax and ventilator-free days highlight the need for further research in this area, alongside the potential need to prioritize technologies for research that are most likely to be clinically effective, cost-effective, safe, and implementable in clinical practice.

We found no evidence that these technologies influence PEEP at a population level, although there was evidence of a small effect on mechanical power and driving pressure. There are two potential reasons for this finding. First, it is possible that while these technologies were used to measure individual patient physiology, the impact on clinical decision-making was limited such that the treating clinician prioritized other clinical data in PEEP titration decisions. Second, it is possible that these technologies informed decisions to both increase and decrease, such that at a population level we observed no difference between groups. This is consistent with the concept that the optimal PEEP level is patent-specific and that personalized strategies are beneficial (14). Setting a patient-specific PEEP may optimize their respiratory mechanics and reduce the risk of harm from invasive mechanical ventilation (19). Although the observed effect on driving pressure and mechanical power was modest, this may still be clinically important as driving pressure and mechanical power are associated with harm and may be the key drivers of ventilator-associated injury (72–74).

Our review has several important limitations. First, few of our included studies were categorized as being at low risk of bias, leading to our categorization of evidence certainty for all assessments as being low or very low. Second, the primary analysis used a pragmatic decision to categorize the evidence by technology, which meant that across studies the same physiologic measure (e.g., transpulmonary pressure) could appear in different groups. This approach was based on the understanding that the safety profile is driven, at least in part, by the technology itself, rather than the physiologic parameters being measured. Third, we identified no relevant evidence in children or any cost-effectiveness studies. Fourth, there was very limited evidence for some technologies, such as nitrogen wash-in/washout measurement of functional residual capacity and ultrasound where only two studies were identified for each. Fifth, pooling the effects across multiple different technologies may obscure the overall effect. However, by reporting in technology subgroup, the impact of each technology can be seen. Finally, many studies did not report key clinical-effectiveness outcomes, with only 10 of the included 22 parallel-group studies reporting 28-day mortality, and just three reporting duration of mechanical ventilation, despite this being a core outcome identified by the ventilation core outcome set (75).

## CONCLUSIONS

Technology-enhanced PEEP optimization strategies do not reduce duration of ventilation, but may reduce mortality. The risk-benefit profile was not consistent and may vary by technology. Overall, evidence was assessed as low- or very low-certainty, highlighting the urgent need for further RCTs on the clinical and cost-effectiveness of these technologies before widespread adoption.

## Supplementary Material
