# Supplementary material for: Technology-Enhanced Strategies to Optimize Positive End-Expiratory Pressure in Patients Receiving Invasive Mechanical Ventilation: A Systematic Review and Meta-Analysis
Source: Crit Care Med. 2026 May 13;54(7):1767–78. doi: 10.1097/CCM.0000000000007144 (PMC13322154; doi:10.1097/CCM.0000000000007144)
Supplement: Supplementary file 1 [file ccm-54-1767-s001.pdf]

Technology-enhanced strategies to optimise positive end expiratory pressure in patients receiving invasive mechanical ventilation: a systematic review and meta-analysis

Supplementary materials

Contents

Record of searching 4

Database searching 4

Grey literature searching 15

Data extraction items 19

Supplementary Table S1: Study characteristics 20

Supplementary Table S2: Study outcomes 22

Supplementary table S3: Categorisation of studies by physiological parameter grouping 25

Supplementary table S4: Sensitivity analyses excluding individual technologies 28

Supplementary table S5: PROGRESS-PLUS items 29

Figure S1 Risk of bias in parallel group randomised trials 31

Figure S2 Risk of bias in included cross-over trials 32

Figure S3: Forest plot- PEEP optimisation strategy v standard care; outcome: Ventilator free days at 28-days 33

Figure S4: Forest plot- PEEP optimisation strategy v standard care; outcome: Intensive care unit length of stay 34

Figure S5: Forest plot- PEEP optimisation strategy v standard care; outcome: Hospital length of stay 35

Figure S6: Forest plot- PEEP optimisation strategy v standard care; outcome: rate of pneumothorax 36

Figure S7: Forest plot- PEEP optimisation strategy v standard care; outcome: rate of barotrauma 37

Figure S8: Forest plot- PEEP optimisation strategy v standard care; outcome: driving pressure 38

Figure S9: Forest plot- PEEP optimisation strategy v standard care; outcome: mechanical power 39

Figure S10: Forest plot- PEEP optimisation strategy v standard care; outcome: Plateau pressure 40

Figure S11: Forest plot- PEEP optimisation strategy v standard care; outcome: Sensitivity analysis by physiological parameter grouping: Duration of mechanical ventilation 41

Figure S12: Forest plot- PEEP optimisation strategy v standard care; outcome: Sensitivity analysis by physiological parameter grouping: 28-day mortality 42

Figure S13: Funnel plot - PEEP optimisation strategy v standard care; outcome: 28 day mortality 43

GRADE tables 44

Record of searching

Database searching

Database Search summary

Database (Platform)	Date searched	Hits	Notes
MEDLINE (Ovid)	25/07/2024	2490	Limited by study type (RCT, SR, economic evaluations) No date or language limits
Embase (Ovid)	25/07/2024	2885	Limited by study type (RCT, SR, economic evaluations) No date or language limits
Science Citation Index and Conference Proceedings – Science (Web of Science)	25/07/2024	1485	Limited by study type (RCT, SR, economic evaluations) No date or language limits
Cochrane Database of Systematic Reviews (Wiley)	26/07/2024	20	No date or language limits
CENTRAL (Wiley)	26/07/2024	1853	No date or language limits
CEA Registry	26/07/2024	71	Used broader scoping review search (based on population terms). No date or language limits.

Total from database searching: 8804

Total after systematically removing 2892 duplicates in EndNote (based on University of Leeds method): 5912

Note: a small number of further duplicates are likely to be identified at the Title/Abstract sifting stage.

MEDLINE (Ovid)

Actual database: Ovid MEDLINE(R) ALL <1946 to July 24, 2024>
Date searched: 25/07/2024

1 ((personalis* or personaliz* or personal or optimis* or optimiz* or optimal or individualis* or individualiz* or individual or impedance tomography* or EIT* or ultrasound* or ultrason* or sono* or echogra* or echotomogra* or echocardiogra* or transpulmonary pressure* or esophageal pressure* or oesophageal pressure* or esophag* manomet* or oesophag* manomet* or compliance* or right heart* or right ventricle* or pressure-volume curve* or P-V curve* or PV curve* or "P/V curve" or "P/V curves" or pressure-volume loop* or P-V loop* or PV loop* or "P/V loop" or "P/V loops" or pressure-volume tool* or P-V tool* or PV tool* or "P/V tool" or "P/V tools" or CT scan* or comput* tomography scan* or CAT scan* or computerised tomography or PaCO2* or ETCO2* or arterial CO2* or end-tidal CO2* or arterial carbon dioxide* or end-tidal carbon dioxide* or partial pressure CO2 or partial pressure of carbon dioxide* or Intellivent?-Adaptive Supportive Ventilation* or Intellivent?-ASV*) adj5 (ventilator* setting* or ventilator* management or PEEP or positive end expiratory pressure or mechanical power or driving pressure)).ti,ab,kf,kw. 1873

2 (personalis* or personaliz* or personal or optimis* or optimiz* or optimal or individualis* or individualiz* or individual or impedance tomography* or EIT* or ultrasound* or ultrason* or sono* or echogra* or echotomogra* or echocardiogra* or transpulmonary pressure* or esophageal pressure* or oesophageal pressure* or esophag* manomet* or oesophag* manomet* or compliance* or right heart* or right ventricle* or pressure-volume curve* or P-V curve* or PV curve* or "P/V curve" or "P/V curves" or pressure-volume loop* or P-V loop* or PV loop* or "P/V loop" or "P/V loops" or pressure-volume tool* or P-V tool* or PV tool* or "P/V tool" or "P/V tools" or CT scan* or comput* tomography scan* or CAT scan* or computerised tomography or PaCO2* or ETCO2* or arterial CO2* or end-tidal CO2* or arterial carbon dioxide* or end-tidal carbon dioxide* or partial pressure CO2 or partial pressure of carbon dioxide* or Intellivent?-Adaptive Supportive Ventilation* or Intellivent?-ASV).ti,ab,kf,kw. 4868659

3 Positive-Pressure Respiration/ 18588

4 2 and 3 5333

5 exp Ultrasonography/ or Manometry/ or Lung Compliance/ or Ventricular Function, Right/ or Ventricular Dysfunction, Right/ or exp Tomography, X-Ray Computed/ 995539

6 3 and 5 1776

7 4 or 6 5980

8 1 or 7 6805

9 exp randomized controlled trial/ 619043

10 controlled clinical trial.pt. 95575

11 randomized.ab. 653833

12 placebo.ab. 250236

13 drug therapy.fs. 2715612

14 randomly.ab. 438280

15 trial.ab. 707166

16 groups.ab. 2709892

17 9 or 10 or 11 or 12 or 13 or 14 or 15 or 16 6019271

18 exp animals/ not humans/ 5241653

19 17 not 18 5268071

20 exp randomized controlled trial/ 619043

21 (random* or "controlled trial*" or "clinical trial*" or rct).tw. 1894246
22 20 or 21 2014513
23 19 or 22 5874154
24 8 and 23 2365
25 Economics/ 27538
26 exp "Costs and Cost Analysis"/ 271894
27 Economics, Nursing/ 4013
28 Economics, Medical/ 9286
29 Economics, Pharmaceutical/ 3143
30 exp Economics, Hospital/ 25912
31 Economics, Dental/ 1922
32 exp "Fees and Charges"/ 31479
33 exp Budgets/ 14233
34 budget*.ti,ab,kf. 38404
35 (economic* or cost or costs or costly or costing or price or prices or pricing or pharmacoeconomic* or pharmaco-economic* or expenditure or expenditures or expense or expenses or financial or finance or finances or financed).ti,kf. 299124
36 (economic* or cost or costs or costly or costing or price or prices or pricing or pharmacoeconomic* or pharmaco-economic* or expenditure or expenditures or expense or expenses or financial or finance or finances or financed).ab. /freq=2 412833
37 (cost* adj2 (effective* or utilit* or benefit* or minimi* or analy* or outcome or outcomes)).ab,kf. 229031
38 (value adj2 (money or monetary)).ti,ab,kf. 3242
39 exp models, economic/ 16423
40 economic model*.ab,kf. 4499
41 markov chains/ 16304
42 markov.ti,ab,kf. 31183
43 monte carlo method/ 33084
44 monte carlo.ti,ab,kf. 63819
45 exp Decision Theory/ 13744
46 (decision* adj2 (tree* or analy* or model*)).ti,ab,kf. 43965
47 or/25-46 [Economic Evaluations & Models - MEDLINE. In: CADTH Search Filters Database. Ottawa: CADTH; 2024: https://searchfilters.cadth.ca/link/16] 958580
48 8 and 47 77
49 (systematic review or meta-analysis).pt. 355506
50 meta-analysis/ or systematic review/ or systematic reviews as topic/ or meta-analysis as topic/ or "meta analysis (topic)"/ or "systematic review (topic)"/ or exp technology assessment, biomedical/ or network meta-analysis/ or exp guideline/ 435370
51 ((systematic* adj3 (review* or overview*)) or (methodologic* adj3 (review* or overview*))).ti,ab,kf. 372782
52 ((quantitative adj3 (review* or overview* or synthes*)) or (research adj3 (integrati* or overview*))).ti,ab,kf. 17782
53 ((integrative adj3 (review* or overview*)) or (collaborative adj3 (review* or overview*)) or (pool* adj3 analy*)).ti,ab,kf. 43227
54 (data synthes* or data extraction* or data abstraction*).ti,ab,kf. 46199
55 (handsearch* or hand search*).ti,ab,kf. 11678
56 (mantel haenszel or peto or der simonian or dersimonian or fixed effect* or latin square*).ti,ab,kf. 38942

57 (met analy* or metanaly* or technology assessment* or HTA or HTAs or technology overview* or technology appraisal*).ti,ab,kf. 13230
58 (meta regression* or metaregression*).ti,ab,kf. 16759
59 (meta-analy* or metaanaly* or systematic review* or biomedical technology assessment* or bio-medical technology assessment*).mp,hw. 529417
60 (medline or cochrane or pubmed or medlars or embase or cinahl).ti,ab,hw. 389536
61 (cochrane or (health adj2 technology assessment) or evidence report).jw. 21974
62 (comparative adj3 (efficacy or effectiveness)).ti,ab,kf. 19342
63 (outcomes research or relative effectiveness).ti,ab,kf. 11839
64 ((indirect or indirect treatment or mixed-treatment or bayesian) adj3 comparison*).ti,ab,kf. 4693
65 (multi* adj3 treatment adj3 comparison*).ti,ab,kf. 311
66 (mixed adj3 treatment adj3 (meta-analy* or metaanaly*)).ti,ab,kf. 181
67 umbrella review*.ti,ab,kf. 2124
68 (multi* adj2 paramet* adj2 evidence adj2 synthesis).ti,ab,kf. 14
69 (multiparamet* adj2 evidence adj2 synthesis).ti,ab,kf. 19
70 (multi-paramet* adj2 evidence adj2 synthesis).ti,ab,kf. 12
71 or/49-70 802206
72 8 and 71 245
73 24 or 48 or 72 2490

Embase (Ovid)

Actual database: Embase Classic+Embase <1947 to 2024 Week 29>
Date searched: 25/07/2024

1 ((personalis* or personaliz* or personal or optimis* or optimiz* or optimal or individualis* or individualiz* or individual or impedance tomography* or EIT* or ultrasound* or ultrason* or sono* or echogra* or echotomogra* or echocardiogra* or transpulmonary pressure* or esophageal pressure* or oesophageal pressure* or esophag* manomet* or oesophag* manomet* or compliance* or right heart* or right ventricle* or pressure-volume curve* or P-V curve* or PV curve* or "P/V curve" or "P/V curves" or pressure-volume loop* or P-V loop* or PV loop* or "P/V loop" or "P/V loops" or pressure-volume tool* or P-V tool* or PV tool* or "P/V tool" or "P/V tools" or CT scan* or comput* tomography scan* or CAT scan* or computerised tomography or PaCO2* or ETCO2* or arterial CO2* or end-tidal CO2* or arterial carbon dioxide* or end-tidal carbon dioxide* or partial pressure CO2 or partial pressure of carbon dioxide* or Intellivent?-Adaptive Supportive Ventilation* or Intellivent?-ASV*) adj5 (ventilator* setting* or ventilator* management or PEEP or positive end expiratory pressure or mechanical power or driving pressure)).ti,ab,kf,kw. 2949
2 (personalis* or personaliz* or personal or optimis* or optimiz* or optimal or individualis* or individualiz* or individual or impedance tomography* or EIT* or ultrasound* or ultrason* or sono* or echogra* or echotomogra* or echocardiogra* or transpulmonary pressure* or esophageal pressure* or oesophageal pressure* or esophag* manomet* or oesophag* manomet* or compliance* or right heart* or right ventricle* or pressure-volume curve* or P-V curve* or PV curve* or "P/V curve" or "P/V curves" or pressure-volume loop* or P-V loop* or PV loop* or "P/V loop" or "P/V loops" or pressure-volume tool* or P-V tool* or PV tool* or "P/V tool" or "P/V tools" or CT scan* or comput* tomography scan* or CAT scan* or computerised tomography or PaCO2* or ETCO2* or arterial CO2* or end-tidal CO2* or arterial carbon dioxide* or end-tidal carbon dioxide* or partial pressure CO2 or partial pressure of

carbon dioxide* or Intellivent?-Adaptive Supportive Ventilation* or Intellivent?-ASV).ti,ab,kf,kw. 6704800
3 exp positive end expiratory pressure ventilation/ 7434
4 2 and 3 2987
5 computer assisted impedance tomography/ or exp echography/ or ultrasound/ or exp esophagus pressure/ or esophagus manometry/ or lung compliance/ or heart right ventricle function/ or pressure volume curve/ or exp computer assisted tomography/ or exp carbon dioxide tension/ 2597229
6 3 and 5 3378
7 1 or 4 or 6 6797
8 exp randomized controlled trial/ 839882
9 controlled clinical trial/ 473992
10 random$.ti,ab. 2113105
11 randomization/ 100082
12 intermethod comparison/ 307882
13 placebo.ti,ab. 386141
14 (compare or compared or comparison).ti,ab. 8229427
15 ((evaluated or evaluate or evaluating or assessed or assess) and (compare or compared or comparing or comparison)).ab. 2977656
16 (open adj label).ti,ab. 117194
17 ((double or single or doubly or singly) adj (blind or blinded or blindly)).ti,ab. 291005
18 double blind procedure/ 224477
19 parallel group$1.ti,ab. 34045
20 (crossover or cross over).ti,ab. 131085
21 ((assign$ or match or matched or allocation) adj5 (alternate or group$1 or intervention$1 or patient$1 or subject$1 or participant$1)).ti,ab. 441381
22 (assigned or allocated).ti,ab. 521951
23 (controlled adj7 (study or design or trial)).ti,ab. 483134
24 (volunteer or volunteers).ti,ab. 297684
25 human experiment/ 668000
26 trial.ti. 440292
27 or/8-26 10784017
28 (random$ adj sampl$ adj7 ("cross section$" or questionnaire$1 or survey$ or database$1)).ti,ab. not (comparative study/ or controlled study/ or randomi?ed controlled.ti,ab. or randomly assigned.ti,ab.) 10204
29 cross-sectional study/ not (exp randomized controlled trial/ or controlled clinical trial/ or controlled study/ or randomi?ed controlled.ti,ab. or control group$1.ti,ab.) 402372
30 (((case adj control$) and random$) not randomi?ed controlled).ti,ab. 22683
31 systematic review.ti,ab. not (trial or study).ti. 366066
32 (nonrandom$ not random$).ti,ab. 19837
33 "random field$".ti,ab. 3100
34 (random cluster adj3 sampl$).ti,ab. 1667
35 (review.ab. and review.pt.) not trial.ti. 1217293
36 "we searched".ab. and (review.ti. or review.pt.) 54176
37 "update review".ab. 143
38 (databases adj4 searched).ab. 70220

39 (rat or rats or mouse or mice or swine or porcine or murine or sheep or lambs or pigs or piglets or rabbit or rabbits or cat or cats or dog or dogs or cattle or bovine or monkey or monkeys or trout or marmoset$1).ti. and animal experiment/ 1263146
40 animal experiment/ not (human experiment/ or human/) 2659643
41 or/28-40 4640931
42 27 not 41 9430884
43 7 and 42 2426
44 Economics/ 249025
45 Cost/ 68324
46 exp Health Economics/ 1105686
47 Budget/ 35466
48 budget*.ti,ab,kf. 51563
49 (economic* or cost or costs or costly or costing or price or prices or pricing or pharmacoeconomic* or pharmaco-economic* or expenditure or expenditures or expense or expenses or financial or finance or finances or financed).ti,kf. 375426
50 (economic* or cost or costs or costly or costing or price or prices or pricing or pharmacoeconomic* or pharmaco-economic* or expenditure or expenditures or expense or expenses or financial or finance or finances or financed).ab. /freq=2 582392
51 (cost* adj2 (effective* or utilit* or benefit* or minimi* or analy* or outcome or outcomes)).ab,kf. 315671
52 (value adj2 (money or monetary)).ti,ab,kf. 4412
53 Statistical Model/ 178654
54 exp economic model/ 4339
55 economic model*.ab,kf.6762
56 Probability/ 160081
57 markov.ti,ab,kf. 41197
58 monte carlo method/ 54413
59 monte carlo.ti,ab,kf. 67845
60 Decision Theory/ 1918
61 Decision Tree/ 25306
62 (decision* adj2 (tree* or analy* or model*)).ti,ab,kf. 59001
63 or/44-62 2169188
64 7 and 63 164
65 meta-analysis/ or systematic review/ or systematic reviews as topic/ or meta-analysis as topic/ or "meta analysis (topic)"/ or "systematic review (topic)"/ or exp technology assessment, biomedical/ or network meta-analysis/ or exp practice guideline/ 1401627
66 ((systematic* adj3 (review* or overview*)) or (methodologic* adj3 (review* or overview*))).ti,ab,kf. 452366
67 ((quantitative adj3 (review* or overview* or synthes*)) or (research adj3 (integrati* or overview*))).ti,ab,kf. 20644
68 ((integrative adj3 (review* or overview*)) or (collaborative adj3 (review* or overview*)) or (pool* adj3 analy*)).ti,ab,kf. 60902
69 (data synthes* or data extraction* or data abstraction*).ti,ab,kf. 56327
70 (handsearch* or hand search*).ti,ab,kf. 14257
71 (mantel haenszel or peto or der simonian or dersimonian or fixed effect* or latin square*).ti,ab,kf. 51741
72 (met analy* or metanaly* or technology assessment* or HTA or HTAs or technology overview* or technology appraisal*).ti,ab,kf. 22554

73 (meta regression* or metaregression*).ti,ab,kf. 20501
74 (meta-analy* or metaanaly* or systematic review* or biomedical technology assessment* or bio-medical technology assessment*).mp,hw. 824811
75 (medline or cochrane or pubmed or medlars or embase or cinahl).ti,ab,hw. 506779
76 (cochrane or (health adj2 technology assessment) or evidence report).jw. 32268
77 (comparative adj3 (efficacy or effectiveness)).ti,ab,kf. 28814
78 (outcomes research or relative effectiveness).ti,ab,kf. 17483
79 ((indirect or indirect treatment or mixed-treatment or bayesian) adj3 comparison*).ti,ab,kf. 8286
80 (multi* adj3 treatment adj3 comparison*).ti,ab,kf. 453
81 (mixed adj3 treatment adj3 (meta-analy* or metaanaly*)).ti,ab,kf. 262
82 umbrella review*.ti,ab,kf. 2246
83 (multi* adj2 paramet* adj2 evidence adj2 synthesis).ti,ab,kf. 35
84 (multiparamet* adj2 evidence adj2 synthesis).ti,ab,kf. 22
85 (multi-paramet* adj2 evidence adj2 synthesis).ti,ab,kf. 30
86 or/65-85 1785840
87 7 and 86 516
88 43 or 64 or 87 2885

Web of Science

Date searched: 25/07/2024

Actual Databases:

- Science Citation Index Expanded (SCI-EXPANDED)–1970-present
- Conference Proceedings Citation Index – Science (CPCI-S)—1990-present

Note: search thread reads from bottom to top.

#16	#15 OR #11 OR #9	1,485
#15	#14 AND #5	154
#14	#13 OR #12	739,203
#13	TS=(metaanalys* or (meta NEAR/1 analys*))	478,598
#12	TS=(systematic* AND (review* OR overview*))	440,672
#11	#10 AND #5	131
#10	TS=(cost* or economic* or pharmacoeconomic* or pharmaco-economic* or price or prices or pricing or expenditure* or expense* or financial or finance or finances or financed or budget* or (value NEAR/1 (money OR monetary)) or (economic NEAR/1 model*) or markov or monte carlo or (decision NEAR/1 (tree* or analy* or model*)))	3,849,725
#9	#8 AND #5	1,348
#8	#7 OR #6	3,016,319
#7	TI=trial	519,768
#6	TS=(random* or "controlled trial*" or "clinical trial*" or RCT or placebo* OR (blind* NEAR/1 (single OR double OR treble OR triple)))	2,887,494

#5	#4 OR #1	4,499
#4	#3 AND #2	3,808
#3	TS=(personalis* OR personaliz* OR personal OR optimis* OR optimiz* OR optimal OR individualis* OR individualiz* OR individual OR "impedance tomography*" OR EIT* OR ultrasound* OR ultrason* OR sono* OR echogra* OR echotomogra* OR echocardiogra* OR "transpulmonary pressure*" OR "esophageal pressure*" OR "oesophageal pressure*" OR "esophag* manomet*" OR "oesophag* manomet*" OR compliance* OR "right heart*" OR "right ventricle*" OR "pressure-volume curve*" OR "P-V curve*" OR "PV curve*" OR "P/V curve" OR "P/V curves" OR "pressure-volume loop*" OR "P-V loop*" OR "PV loop*" OR "P/V loop" OR "P/V loops" OR "pressure-volume tool*" OR "P-V tool*" OR "PV tool*" OR "P/V tool" OR "P/V tools" OR "CT scan*" OR "comput* tomography scan*" OR "CAT scan*" OR "computerised tomography" OR PaCO2* OR ETCO2* OR "arterial CO2*" OR "end-tidal CO2*" OR "arterial carbon dioxide*" OR "end-tidal carbon dioxide*" OR "partial pressure CO2" OR "partial pressure of carbon dioxide*" OR "Intellivent$-Adaptive Supportive Ventilation*" OR Intellivent$-ASV*)	9,234,702
#2	TS=(positive end expiratory pressure)	7,745
#1	TS=((personalis* OR personaliz* OR personal OR optimis* OR optimiz* OR optimal OR individualis* OR individualiz* OR individual OR "impedance tomography*" OR EIT* OR ultrasound* OR ultrason* OR sono* OR echogra* OR echotomogra* OR echocardiogra* OR "transpulmonary pressure*" OR "esophageal pressure*" OR "oesophageal pressure*" OR "esophag* manomet*" OR "oesophag* manomet*" OR compliance* OR "right heart*" OR "right ventricle*" OR "pressure-volume curve*" OR "P-V curve*" OR "PV curve*" OR "P/V curve" OR "P/V curves" OR "pressure-volume loop*" OR "P-V loop*" OR "PV loop*" OR "P/V loop" OR "P/V loops" OR "pressure-volume tool*" OR "P-V tool*" OR "PV tool*" OR "P/V tool" OR "P/V tools" OR "CT scan*" OR "comput* tomography scan*" OR "CAT scan*" OR "computerised tomography" OR PaCO2* OR ETCO2* OR "arterial CO2*" OR "end-tidal CO2*" OR "arterial carbon dioxide*" OR "end-tidal carbon dioxide*" OR "partial pressure CO2" OR "partial pressure of carbon dioxide*" OR "Intellivent$-Adaptive Supportive Ventilation*" OR Intellivent$-ASV*) NEAR/5 ("ventilator* setting*" OR "ventilator* management" OR PEEP OR "positive end expiratory pressure" OR "mechanical power" OR "driving pressure"))	1,892

Total: 1485

Cochrane Library

Date searched: 26/07/2024

ID Search Hits

#1	((personalis* OR personaliz* OR personal OR optimis* OR optimiz* OR optimal OR individualis* OR individualiz* OR individual OR (impedance NEXT tomography*) OR EIT* OR ultrasound* OR ultrason* OR sono* OR echogra* OR echotomogra* OR echocardiogra* OR (transpulmonary NEXT pressure*) OR (esophageal NEXT pressure*) OR (oesophageal NEXT pressure*) OR (esophag* NEXT manomet*) OR (oesophag* NEXT manomet*) OR compliance* OR (right NEXT heart*) OR (right NEXT ventricle*) OR (pressure-volume NEXT curve*) OR ("P-V" NEXT curve*) OR ("PV" NEXT curve*) OR "P/V curve" OR "P/V curves" OR ("pressure-volume" NEXT loop*) OR ("P-V" NEXT loop*) OR ("PV" NEXT loop*) OR "P/V loop" OR "P/V loops" OR ("pressure-volume" NEXT tool*) OR ("P-V" NEXT tool*) OR ("PV" NEXT tool*) OR "P/V tool" OR "P/V tools" OR ("CT" NEXT scan*) OR (comput* NEXT "tomography" NEXT scan*) OR ("CAT" NEXT scan*) OR "computerised tomography" OR PaCO2* OR ETCO2* OR ("arterial" NEXT CO2*) OR ("end-tidal" NEXT CO2*) OR ("arterial carbon" NEXT dioxide*) OR ("end-tidal carbon" NEXT dioxide*) OR "partial pressure CO2" OR ("partial pressure of carbon" NEXT dioxide*) OR (Intellivent* NEXT ("ASV" OR "Adaptive Supportive Ventilation"))) NEAR/5 ((ventilator* NEXT setting*) OR (ventilator* NEXT "management") OR PEEP OR "positive end expiratory pressure" OR "mechanical power" OR "driving pressure")):ti,ab,kw	966

#2	(personalis* OR personaliz* OR personal OR optimis* OR optimiz* OR optimal OR individualis* OR individualiz* OR individual OR ("impedance" NEXT tomography*) OR EIT* OR ultrasound* OR ultrason* OR sono* OR echogra* OR echotomogra* OR echocardiogra* OR ("transpulmonary" NEXT pressure*) OR ("esophageal" NEXT pressure*) OR ("oesophageal" NEXT pressure*) OR (esophag* NEXT manomet*) OR (oesophag* NEXT manomet*) OR compliance* OR ("right" NEXT heart*) OR ("right" NEXT ventricle*) OR ("pressure-volume" NEXT curve*) OR ("P-V" NEXT curve*) OR ("PV" NEXT curve*) OR "P/V curve" OR "P/V curves" OR ("pressure-volume" NEXT loop*) OR ("P-V" NEXT loop*) OR ("PV" NEXT loop*) OR "P/V loop" OR "P/V loops" OR ("pressure-volume" NEXT tool*) OR ("P-V" NEXT tool*) OR ("PV" NEXT tool*) OR "P/V tool" OR "P/V tools" OR ("CT" NEXT scan*) OR (comput* NEXT "tomography" NEXT scan*) OR ("CAT" NEXT scan*) OR "computerised tomography" OR PaCO2* OR ETCO2* OR ("arterial" NEXT CO2*) OR ("end-tidal" NEXT CO2*) OR ("arterial carbon" NEXT dioxide*) OR ("end-tidal carbon" NEXT dioxide*) OR "partial pressure CO2" OR ("partial pressure of carbon" NEXT dioxide*) OR (Intellivent* NEXT ("ASV" OR "Adaptive Supportive Ventilation"))):ti,ab,kw	580828

#3	[mh ^"Positive-Pressure Respiration"]	2122

#4	#2 AND #3	1148

#5	[mh Ultrasonography] OR [mh ^Manometry] OR [mh ^"Lung Compliance"] OR [mh ^"Ventricular Function, Right"] OR [mh ^"Ventricular Dysfunction, Right"] OR [mh "Tomography, X-Ray Computed"]	29490

#6	#3 AND #5	192

#7	#4 OR #6	1160

#8	#1 OR #7	1873

CDSR:

Reviews:	20

Protocols:	0

CENTRAL Total:	1853

CEA Registry

https://cear.tuftsmedicalcenter.org/

Date searched: 15 October 2023 (updated in May 2024 and July 2024)

Note: Our broader scoping review search used population terms. For efficiency in this small database, we decided to update our scoping review search (with no date limit) for this and our other related systematic reviews rather than develop 4 new searches. We cross-checked any records originally selected as potentially relevant for the scoping review and all new records against the eligibility criteria of each systematic review.

Advanced search

Keyword is ventilation
OR
Keyword is ventilate
OR
Keyword is ventilates
OR
Keyword is ventilated
OR
Keyword is ventilator
OR
Keyword is ventilators
OR
Keyword is intubation
OR
Keyword is intubate
OR
Keyword is intubates
OR
Keyword is intubated
OR
Keyword is re-intubation
OR
Keyword is re-intubate
OR
Keyword is re-intubates
OR
Keyword is extubation
OR
Keyword is extubate
OR
Keyword is extubates
OR
Keyword is extubated
OR
Keyword is post-extubation
OR
Keyword is acute respiratory distress syndrome

OR
Keyword is ARDS
OR
Keyword is respiratory failure
OR
Keyword is AHRF
OR
Keyword is artificial respiration
OR
Keyword is artificial respirator
OR
Keyword is artificial respirators
OR
Keyword is IMV

Total: 69
Downloaded to Excel. Screened in Excel by reviewer.

Update May 2024

Note: search interface changed.

Search date: 22/05/2024

Advanced search

keyword:("ventilation" OR "ventilate" OR "ventilates" OR "ventilated" OR "ventilator" OR "ventilators" OR "intubation" OR "intubate" OR "intubates" OR "intubated" OR "re-intubation" OR "re-intubate" OR "re-intubates" OR "extubate" OR "extubates" OR "extubated" OR "post-extubation" OR "acute respiratory distress syndrome" OR "ARDS" OR "respiratory failure" OR "AHRF" OR "ARDS" OR "artificial respiration" OR "artificial respirator" OR "artificial respirators" OR "IMV")

Total: 70
Total after duplicates with original CEA Registry search removed: 4
Additional 4 Downloaded to Excel.
Total after duplicates with original searches removed: 2
Screened in Excel by information specialist - not relevant (title 1: The cost-effectiveness of standalone HEPA filtration units for the prevention of airborne SARS CoV-2 transmission. Title 2: A Cost-Utility Analysis of Remote Pulse-Oximetry Monitoring of Patients With COVID-19.)

Update July 2024

Search date: 26/07/2024

Advanced search

keyword:("ventilation" OR "ventilate" OR "ventilates" OR "ventilated" OR "ventilator" OR "ventilators" OR "intubation" OR "intubate" OR "intubates" OR "intubated" OR "re-intubation" OR "re-intubate" OR "re-intubates" OR "extubate" OR "extubates" OR "extubated" OR "post-extubation" OR "acute respiratory distress syndrome" OR "ARDS" OR "respiratory failure" OR "AHRF" OR "ARDS" OR "artificial respiration" OR "artificial respirator" OR "artificial respirators" OR "IMV")

Total: 71
Total after duplicates with previous CEA Registry search removed: 1
Additional 1 Downloaded to Excel.
Total after duplicates with original searches removed: 1
Screened in Excel by information specialist - not relevant (title 1: Cost-effectiveness of home non-invasive ventilation in patients with persistent hypercapnia after an acute exacerbation of COPD in the UK.)

Grey literature searching

Dissertations and Theses

Dissertations & Theses Global (ProQuest)

Date searched: 14/11/2024

(TI,AB((personalis* OR personaliz* OR personal OR optimis* OR optimiz* OR optimal OR individualis* OR individualiz* OR individual OR "impedance tomography" OR EIT* OR ultrasound* OR ultrason* OR sono* OR echogra* OR echotomogra* OR echocardiogra* OR "transpulmonary pressure*" OR "esophageal pressure*" OR "oesophageal pressure*" OR (esophag* NEAR/3 manomet*) OR (oesophag* NEAR/3 manomet*) OR compliance* OR "right heart" OR "right ventricle" OR "pressure-volume curve*" OR "P-V curve*" OR "PV curve*" OR "P/V curve*" OR "pressure-volume loop*" OR "P-V loop*" OR "PV loop*" OR "P/V loop*" OR "pressure-volume tool*" OR "P-V tool*" OR "PV tool*" OR "P/V tool*" OR "CT scan*" OR "computer tomography scan*" OR "CAT scan*" OR "computerised tomography" OR PaCO2 OR ETCO2 OR "arterial CO2" OR "end-tidal CO2" OR "arterial carbon dioxide" OR "end-tidal carbon dioxide" OR "partial pressure CO2" OR "partial pressure of carbon dioxide" OR "Intellivent?-Adaptive Supportive Ventilation*" OR Intellivent?-ASV*) AND ((ventilat* NEAR/1 (setting* OR management)) OR "PEEP" OR "positive end expiratory pressure" OR "mechanical power" OR "driving pressure")))

Total: 397

Applied filters

NOT (animals AND aerospace engineering AND alternative energy AND civil engineering AND industrial engineering AND zoology AND mechanical engineering AND electrical engineering):

Total: 253

After information specialist filtered out animal studies and studies in different disciplines and topics:

Total: 50 (sent to reviewer for checking)

After reviewer screened titles and abstracts:

Total: 2

After reviewer screened full text:

Total: 0

Trial registers

ClinicalTrials.gov

Date searched: 21/11/2024

Other terms: ((personalise OR personal OR optimise OR optimal OR individualise OR individual OR "impedance tomography" OR EIT OR ultrasound OR ultrasonography OR sonography OR echography OR echotomography OR echocardiography OR "transpulmonary pressure" OR "esophageal pressure" OR "oesophageal pressure" OR "esophageal manometry" OR "oesophageal manometry" OR compliance OR "right heart" OR "right ventricle" OR (("pressure volume" OR PV OR "P/V" OR "P-V") AND (curve OR loop OR tool)) OR "CT scan" OR "computer tomography scan" OR "CAT scan" OR "computerised tomography" OR "PaCO2" OR "ETCO2" OR "arterial CO2" OR "end-tidal CO2" OR "arterial carbon dioxide" OR "end-tidal carbon dioxide" OR "partial pressure CO2" OR "partial pressure of carbon dioxide" OR (Intellivent AND "Adaptive Supportive Ventilation") OR (Intellivent AND ASV)) AND ("ventilator setting" OR "ventilator management" OR PEEP OR "positive end expiratory pressure" OR "mechanical power" OR "driving pressure")) | Studies with results

Total: 41

Total after information specialist filtered out 37 irrelevant or duplicate records from other searches: 4

Total after information specialist found related publications from database searches: 1 (sent to reviewer)

Note: Other trial register records for ongoing, terminated and completed trials were found via CENTRAL for screening by reviewer in EndNote.

Websites

Manufacturers' websites

**Source(s)**	**Date searched**	**Description of search**	**Hits**	**Notes**
Drager- https://www.draeger.com/en_uk/Hospital/Electrical-Impedance-Tomography#clinical-evidence	21/11/2024	Browsed website. In sub-section of Electrical-Impedance-Tomography#clinical-evidence headed 'Scientific publications on EIT', the following PDFs were found		Information specialist cross-checked references PDFs against records found in our previous database searches.<br><br>Reviewer checked Title and Abstract of newly identified records.
		Literature List 2019	Total: 23<br>• 8 already found in database searches<br>• 15 sent for reviewer check	
		Literature List 2020	Total: 38<br>• 15 already found in database searches (1 in included studies list)<br>• 23 sent for reviewer check	
		Literature List 2021	Total: 44<br>• 15 already found in database searches (1 in included studies list)	

			• 29 sent for reviewer check	
		Literature List 2022	Total: 41<br>• 11 already found in database searches<br>• 30 sent for reviewer check	
Sentec<br>https://www.sentec.com/acute-respiratory-care/	21/11/2024	Browsed website.<br>'Electrical Impedance Tomography (EIT)' and<br>'EIT IN ACUTE RESPIRATORY CARE'<br><br>Identified PDF: " Electrical Impedance Tomography (EIT) in Adult Critical Care: A Curated Bibliography"<br>Sections:<br>'Select Reviews and Consensus guidelines' and<br>'Personalization of Ventilation'	Total: 16<br>• 13 already found in database searches<br>• 3 sent for reviewer check	

Data extraction items

Study level characteristics: Author, Year, Title, Country and continent, Study design

Population level characteristics: Sample size, Age, Sex, other PROGRESS-PLUS related characteristics (place of residence, race/ethnicity culture/language, occupation, religion, education, socio-economic status and social capital, sexual orientation and disability)

Intervention and comparator level characteristics: Description of intervention, description of comparator

Outcome level characteristics: List of outcomes, outcome events and group total, outcome mean (standard deviation) and group total.

Supplementary Table S1: Study characteristics

ARDS: Acute Respiratory Distress Syndrome; FRC: Functional residual capacity; IAP: Intra-abdominal pressure; ICU: Intensive Care Unit; PEEP: Positive end-expiratory pressure; RCT: Randomised controlled trial; TPP: transpulmonary pressure; VV-ECMO: Veno-venous extracorporeal membrane oxygenation

Study Name	Setting and Design	Population	Intervention	Comparator
Antonin 2022	France. Single centre cross-over RCT	20 adults – ARDS	Electrical impedance tomography	Standard care
Costa 2024	Brazil. Single centre RCT	76 adults – ARDS	Electrical impedance tomography	Standard care
He 2021	China. Single centre RCT	117 adults – ARDS	Electrical impedance tomography	Standard care
Hsu 2021	Taiwan. Single centre RCT	87 adults – ARDS	Electrical impedance tomography	Pressure-volume curve analysis
Jimenez 2023	USA. Single centre crossover-RCT	16 adults – ARDS	Electrical impedance tomography	Standard care
Mauri 2024	Italy. Multi-centre crossover-RCT	21 adults – ARDS	Electrical impedance tomography	Standard care
Scaramuzzo 2020	Italy. Multi-centre crossover-RCT	20 adults – ARDS	Electrical impedance tomography	Oesophageal balloon (TPP)
Arnal 2012	France. Single centre crossover-RCT	50 adults – mixed	Fully automated closed loop ventilation	Standard care
Buiteman-Kruizinga 2023	Netherlands. Single centre crossover-RCT	13 adults – ARDS	Fully automated closed loop ventilation	Standard care
Chelly 2020	France. Single centre crossover-RCT	265 adults – mixed	Fully automated closed loop ventilation	Standard care
Komnov 2023	Russia. Single centre RCT	32 adults – BMI >35, post cardiac surgery	Fully automated closed loop ventilation	Standard care
Yeremenko 2022	Russia. Single centre RCT	120 adults – post cardiac surgery	Fully automated closed loop ventilation	Standard care
Salem 2020	Egypt. Single centre RCT	60 adults – ARDS	Lung ultrasound	Standard care
Tang 2017	China. Single centre RCT	40 adults – ARDS	Lung ultrasound	Standard care
Di 2020	China. Single centre RCT	78 adults – ARDS	Nitrogen wash-in/washout (FRC)	Standard care
Rollas 2022	Turkey. Single centre RCT	22 adults – ARDS	Nitrogen wash-in/washout (FRC)	Standard care

Beitler 2019	USA and Canada. Multi-centre RCT	200 adults – ARDS	Oesophageal balloon (TPP)	Standard care
Bergez 2019	France. Multi-centre crossover-RCT	19 adults – ARDS	Oesophageal balloon (TPP)	Standard care
Dara 2022	France. Single centre crossover-RCT	20 adults – ARDS	Oesophageal balloon (TPP - end expiratory)	Oesophageal balloon (TPP - end inspiratory)
Li 2016	China. Single centre RCT	35 adults – (pancreatitis with increased IAP)	Oesophageal balloon (TPP)	Standard care
Obi 2018	USA. Single centre RCT	25 adults – BMI ≥40	Oesophageal balloon (TPP)	Static respiratory compliance
Talmor 2008	USA. Single centre RCT	61 adults – ARDS	Oesophageal balloon (TPP)	Standard care
Wang 2019	China. Single centre RCT	23 adults – ARDS	Oesophageal balloon (TPP)	Standard care
Wang 2020	China. Single centre RCT	104 adults – ARDS (VV-ECMO)	Oesophageal balloon (TPP)	Standard care
Wu 2016	China. Single centre crossover-RCT	28 adults – ARDS	Oesophageal balloon (TPP)	Standard care
Yang 2013	China. Single centre crossover-RCT	15 adults – ARDS	Oesophageal balloon (TPP)	Standard care
Amato 1998	Brazil. Multi-centre RCT	53 adults – ARDS	Pressure-volume curve	Standard care
Hu 2024	China. Single centre RCT	81 adults – ARDS	Pressure-volume curve	Standard care
Huang 2013	China. Single centre crossover-RCT	30 adults – ARDS	Pressure-volume curve	Standard care
Long 2006	China. Single centre RCT	30 adults – ARDS	Pressure-volume curve	Standard care
Nguyen 2019	Vietnam. Single centre RCT	40 adults – ARDS	Pressure-volume curve analysis	Standard care
Cavalcanti 2017	Multi-national (Europe/South America). Multi-centre RCT	1010 adults – ARDS	Static respiratory compliance	Standard care
Pintado 2013	Spain. Single centre RCT	70 adults – ARDS	Static respiratory compliance	Standard care
Pintado 2017	Spain. Single centre RCT	70 adults – ARDS	Static respiratory compliance	Standard care

Supplementary Table S2: Study outcomes

Study Name	Measured Outcomes
Antonin 2022	- PEEP
Costa 2024	- Mortality (28 day)<br>- Barotrauma
He 2021	- Mortality (28 day)<br>- Ventilator free days (at 28 days)<br>- ICU length of stay<br>- PEEP<br>- Driving pressure
Hsu 2021	- Survival to hospital discharge<br>- Duration of mechanical ventilation<br>- ICU length of stay<br>- Barotrauma<br>- PEEP<br>- Driving pressure
Jimenez 2023	- PEEP<br>- Driving pressure<br>- Plateau pressure<br>- Peak pressure<br>- Mechanical power
Mauri 2024	- PEEP
Scaramuzzo 2020	- PEEP<br>- Driving pressure<br>- Mechanical power
Arnal 2012	- PEEP<br>- Plateau pressure<br>- Peak pressure
Buiteman-Kruizinga 2023	- PEEP<br>- Plateau pressure<br>- Pneumothorax
Chelly 2020	- PEEP<br>- Peak pressure
Komnov 2023	- Duration of mechanical ventilation<br>- PEEP<br>- Driving pressure<br>- Mechanical power
Yeremenko 2022	- Mortality (hospital)<br>- Duration of mechanical ventilation<br>- ICU length of stay<br>- Hospital length of stay<br>- PEEP<br>- Driving pressure<br>- Mechanical power
Salem 2020	- Mortality (28 day)<br>- Duration of mechanical ventilation

	- Ventilator free days (at 28 days)<br>- ICU length of stay<br>- Barotrauma
Tang 2017	- PEEP<br>- Peak pressure
Di 2020	- Mortality (28 day)<br>- PEEP<br>- Mechanical power
Rollas 2022	- PEEP<br>- Driving pressure
Beitler 2019	- Mortality (28 days, 60 days, 1 year)<br>- Ventilator free days (at 28 days)<br>- ICU length of stay (at 28 days, 60 days)<br>- Hospital length of stay (at 28 days, 60 days)<br>- Pneumothorax<br>- Barotrauma
Bergez 2019	- PEEP<br>- Plateau pressure<br>- Peak pressure<br>- Pneumothorax
Dara 2022	- PEEP
Li 2016	- PEEP
Obi 2018	- Duration of mechanical ventilation<br>- Weaning success at 30 days<br>- PEEP
Talmor 2008	- Mortality (28 day)<br>- Ventilator free days (at 28 days)<br>- ICU length of stay<br>- PEEP<br>- Plateau pressure<br>- Peak pressure<br>- Mean airway pressure
Wang 2019	- PEEP<br>- Peak pressure
Wang 2020	- Mortality (60 day, 6 months)<br>- Ventilator free days (at 28 days)<br>- ICU length of stay<br>- Hospital length of stay<br>- Barotrauma<br>- PEEP<br>- Driving pressure<br>- Peak pressure<br>- Mechanical power
Wu 2016	- PEEP<br>- Plateau pressure
Yang 2013	- PEEP<br>- Plateau pressure

Amato 1998	- Mortality (28 day, ICU, hospital)
Hu 2024	- Mortality (28 day)<br>- Duration of mechanical ventilation<br>- ICU length of stay<br>- Peak pressure
Huang 2013	- Plateau pressure<br>- Peak pressure<br>- Mean airway pressure
Long 2006	- Mortality (28 day)<br>- PEEP<br>- Plateau pressure
Nguyen 2019	- Mortality (28 day)<br>- PEEP
Cavalcanti 2017	- Mortality (28 day, 6 month, ICU, hospital)<br>- Ventilator free days (at 28 days)<br>- ICU length of stay<br>- Hospital length of stay<br>- Pneumothorax requiring drainage within 7 days<br>- Barotrauma within 7 days
Pintado 2013	- Mortality (28 day)<br>- Ventilator free days (at 28 days)<br>- ICU length of stay<br>- Barotrauma
Pintado 2017	- Mortality (28 day, 90 days)<br>- Ventilator free days (at 28 days)<br>- Barotrauma

Supplementary table S3: Categorisation of studies by physiological parameter grouping

Physiological Parameter	Technologies	Key Metrics
Regional ventilation & aeration	Electrical Impedance Tomography, Lung Ultrasound	Regional ventilation, aeration, collapse/overdistension
Lung volume	Nitrogen Wash-in/Wash-out	FRC
Respiratory mechanics	Oesophageal Balloon, Pressure–Volume Curve, Static Compliance	Pleural pressure, transpulmonary pressure, compliance
Gas exchange & control	Fully Automated Closed-Loop Ventilation	SpO_2, $ETCO_2$, resistance, compliance

Study Name	Intervention	Comparator	Physiological Parameter
Beitler 2019	Oesophageal balloon (transpulmonary pressure)	Standard care	Respiratory mechanics
Bergez 2019	Oesophageal balloon (transpulmonary pressure)	Standard care	Respiratory mechanics
Dara 2022	Oesophageal balloon (transpulmonary pressure - end expiratory)	Oesophageal balloon (transpulmonary pressure - end inspiratory)	Respiratory mechanics
Li 2016	Oesophageal balloon (transpulmonary pressure)	Standard care	Respiratory mechanics
Obi 2018	Oesophageal balloon (transpulmonary pressure)	Static respiratory compliance	Respiratory mechanics
Talmor 2008	Oesophageal balloon (transpulmonary pressure)	Standard care	Respiratory mechanics

Wang 2019	Oesophageal balloon (transpulmonary pressure)	Standard care	Respiratory mechanics
Wang 2020	Oesophageal balloon (transpulmonary pressure)	Standard care	Respiratory mechanics
Wu 2016	Oesophageal balloon (transpulmonary pressure)	Standard care	Respiratory mechanics
Yang 2013	Oesophageal balloon (transpulmonary pressure)	Standard care	Respiratory mechanics
Amato 1998	Pressure-volume curve analysis	Standard care	Respiratory mechanics
Hu 2024	Pressure-volume curve analysis	Standard care	Respiratory mechanics
Huang 2013	Pressure-volume curve analysis	Standard care	Respiratory mechanics
Long 2006	Pressure-volume curve analysis	Standard care	Respiratory mechanics
Nguyen 2019	Pressure-volume curve analysis	Standard care	Respiratory mechanics
Cavalcanti 2017	Static respiratory compliance	Standard care	Respiratory mechanics
Pintado 2013	Static respiratory compliance	Standard care	Respiratory mechanics
Pintado 2017	Static respiratory compliance	Standard care	Respiratory mechanics
Hsu 2021	Electrical impedance tomography	Pressure-volume curve analysis	Regional ventilation & aeration<br>Respiratory mechanics
Antonin 2022	Electrical impedance tomography	Standard care	Regional ventilation & aeration

Costa 2024	Electrical impedance tomography	Standard care	Regional ventilation & aeration
He 2021	Electrical impedance tomography	Standard care	Regional ventilation & aeration
Jimenez 2023	Electrical impedance tomography	Standard care	Regional ventilation & aeration
Mauri 2024	Electrical impedance tomography	Standard care	Regional ventilation & aeration
Scaramuzzo 2020	Electrical impedance tomography	Oesophageal balloon (transpulmonary pressure)	Regional ventilation & aeration
Salem 2020	Lung ultrasound	Standard care	Regional ventilation & aeration
Tang 2017	Lung ultrasound	Standard care	Regional ventilation & aeration
Arnal 2012	Fully automated closed loop ventilation	Standard care	Gas exchange & control
Buiteman-Kruizinga 2023	Fully automated closed loop ventilation	Standard care	Gas exchange & control
Chelly 2020	Fully automated closed loop ventilation	Standard care	Gas exchange & control
Komnov 2023	Fully automated closed loop ventilation	Standard care	Gas exchange & control
Yeremenko 2022	Fully automated closed loop ventilation	Standard care	Gas exchange & control
Di 2020	Nitrogen wash-in/washout (functional residual capacity)	Standard care	Lung volume
Rollas 2022	Nitrogen wash-in/washout (functional residual capacity)	Standard care	Lung volume

Supplementary table S4: Sensitivity analyses excluding individual technologies

	**Mortality**	
	Studies (patients) in analysis	Risk Ratio (95% CI)
Overall analysis	10 studies (1,719 patients)	0.69, 95% CI 0.52 to 0.93
Exclude pressure volume curve	7 studies (1,596 patients)	0.72, 95% CI 0.50 to 1.04
Exclude Oesophageal balloon (transpulmonary pressure)	8 studies (1,458 patients)	0.66, 95% CI 0.46 to 0.94
Exclude Respiratory static compliance	8 studies (639 patients)	0.66, 95% CI 0.50 to 0.87
Exclude Nitrogen multiple breath washout/wash-in	9 studies (1,641 patients)	0.73, 95% CI 0.54 to 0.98
Exclude Electrical impedance tomography	9 studies (1,602 patients)	0.68, 95% CI 0.49 to 0.93
Exclude Lung ultrasound	9 studies (1,659 patients)	0.73, 95% CI 0.55 to 0.97
	**Duration of mechanical ventilation**	
	Studies (patients) in analysis	Mean difference (95% CI)
Overall analysis	3 studies (172 patients)	-0.06, 95% CI -0.20 to 0.09
Exclude Intellivent-ASV	1 study (60 patients)	-3.90, 95% CI -5.58 to -2.22
Exclude Lung ultrasound	2 studies (112 patients)	-0.04, 95% CI -0.05 to -0.02

Supplementary table S5: PROGRESS-PLUS items

	Place of residence	Race/ ethnicity	Occupation	Gender	Sex	Religion	Education	Socioeconomic status	Social capital	Personal characteristics associated with discrimination	Features of relationships	Time-dependent relationships
Amato 1998	X	X	X	X	X	X	X	X	X	X	X	X
Antonin 2022	X	X	X	X	X	X	X	X	X	X	X	X
Arnal 2012	X	X	X	X	✓	X	X	X	X	X	X	X
Beitler 2019	X	X	X	X	✓	X	X	X	X	X	X	X
Bergez 2019	X	X	X	X	✓	X	X	X	X	X	X	X
Buiteman-Kruizinga 2023	X	X	X	X	✓	X	X	X	X	X	X	X
Cavalcanti 2017	X	X	X	X	✓	X	X	X	X	X	X	X
Chelly 2020	X	X	X	X	✓	X	X	X	X	X	X	X
Costa 2024	X	X	X	X	✓	X	X	X	X	X	X	X
Dara 2022	X	X	X	X	X	X	X	X	X	X	X	X
Di 2020	X	X	X	X	✓	X	X	X	X	X	X	X
He 2021	X	X	X	X	✓	X	X	X	X	X	X	X
Hsu 2021	X	X	X	X	✓	X	X	X	X	X	X	X
Hu 2024	X	X	X	X	X	X	X	X	X	X	X	X
Huang 2013	X	X	X	X	✓	X	X	X	X	X	X	X
Jimenez 2023	X	✓	X	X	✓	X	X	X	X	X	X	X

Komnov 2023	X	X	X	X	X	X	X	X	X	X	X	X
Li 2016	X	X	X	X	X	X	X	X	X	X	X	X
Long 2006	X	X	X	X	X	X	X	X	X	X	X	X
Mauri 2024	X	X	X	X	X	X	X	X	X	X	X	X
Nguyen 2019	X	X	X	X	X	X	X	X	X	X	X	X
Obi 2018	X	X	X	X	✓	X	X	X	X	X	X	X
Pintado 2013	X	X	X	X	✓	X	X	X	X	X	X	X
Pintado 2017	X	X	X	X	✓	X	X	X	X	X	X	X
Rollas 2022	X	X	X	X	✓	X	X	X	X	X	X	X
Salem 2020	X	X	X	X	✓	X	X	X	X	X	X	X
Scaramuzzo 2020	X	X	X	X	✓	X	X	X	X	X	X	X
Talmor 2008	X	✓	X	X	✓	X	X	X	X	X	X	X
Tang 2017	X	X	X	X	✓	X	X	X	X	X	X	X
Wang 2019	X	X	X	X	✓	X	X	X	X	X	X	X
Wang 2020	X	X	X	X	✓	X	X	X	X	X	X	X
Wu 2016	X	X	X	X	✓	X	X	X	X	X	X	X
Yang 2013	X	X	X	X	✓	X	X	X	X	X	X	X
Yeremenko 2022	X	X	X	X	✓	X	X	X	X	X	X	X

Figure S1 Risk of bias in parallel group randomised trials

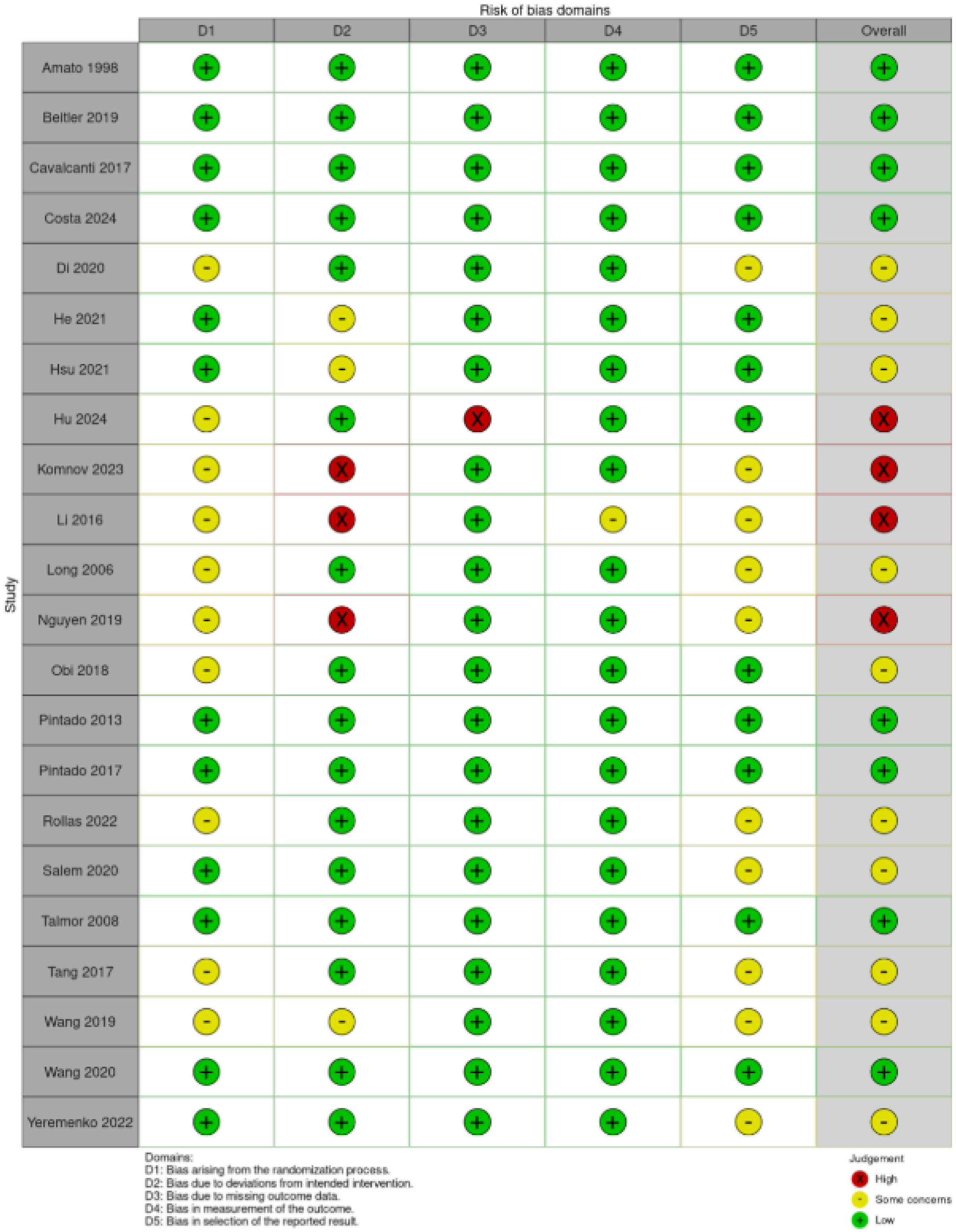

Figure S2 Risk of bias in included cross-over trials

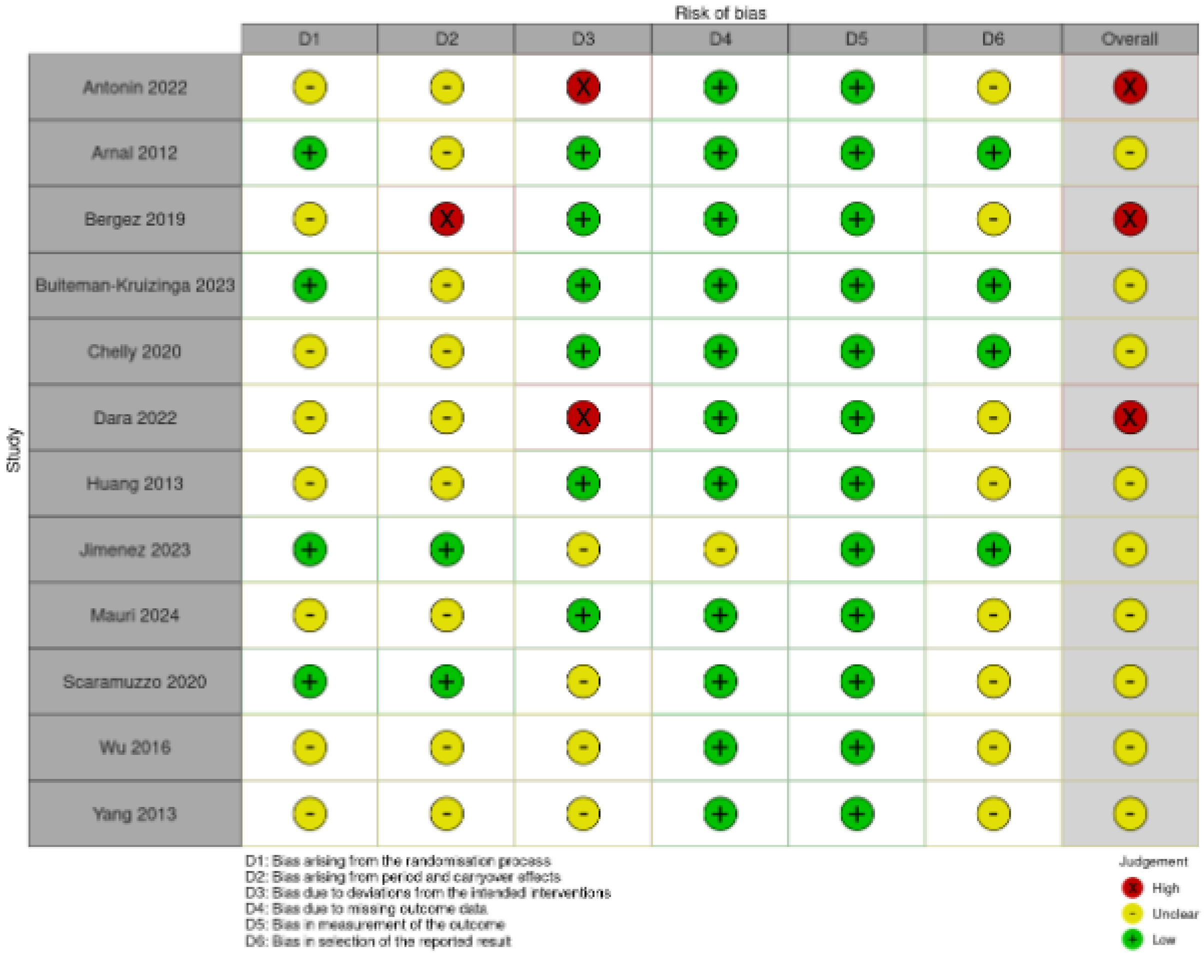

Figure S3: Forest plot- PEEP optimisation strategy v standard care; outcome: Ventilator free days at 28-days

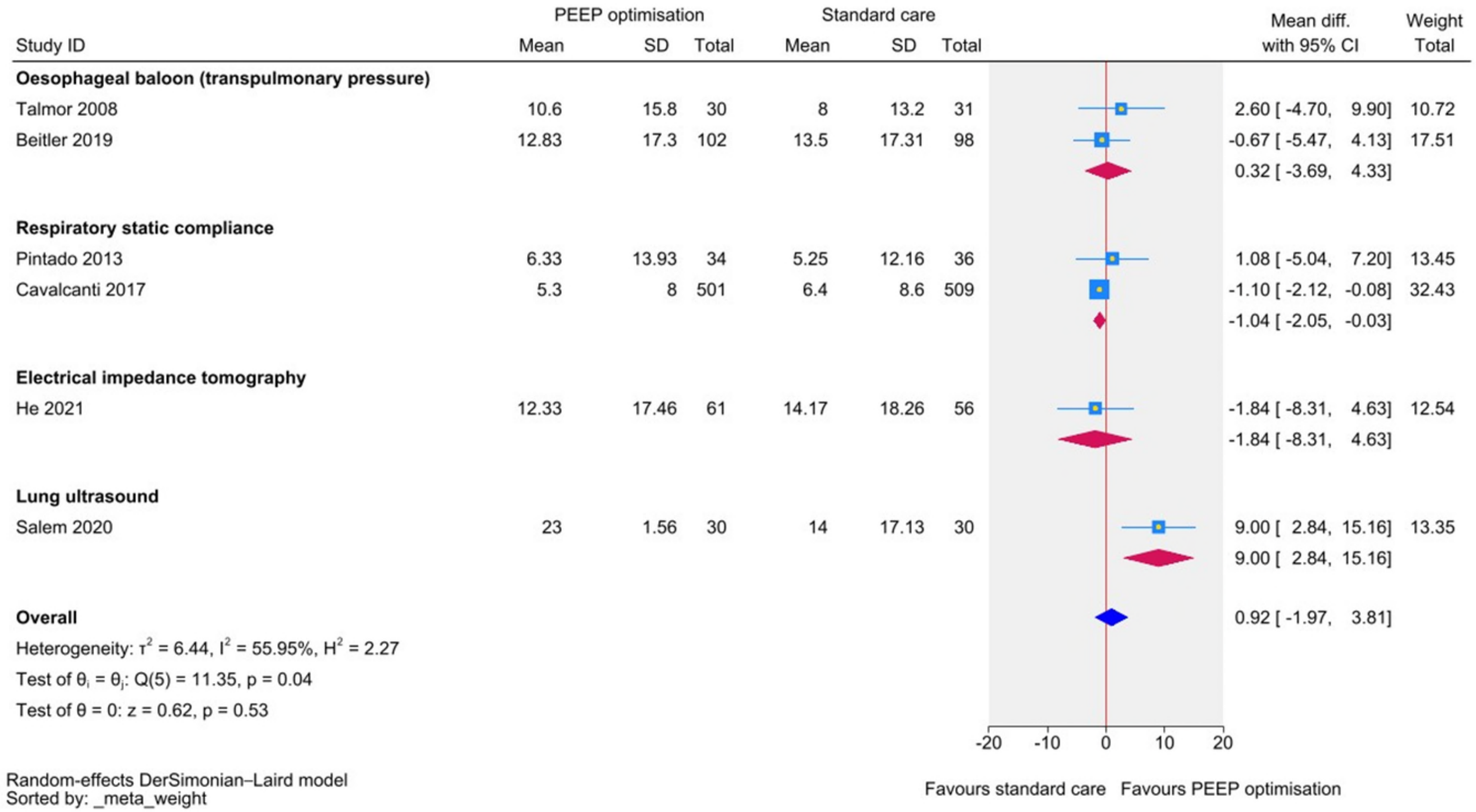

Figure S4: Forest plot- PEEP optimisation strategy v standard care; outcome: Intensive care unit length of stay

Study ID	PEEP optimisation Mean	PEEP optimisation SD	PEEP optimisation Total	Standard care Mean	Standard care SD	Standard care Total	Mean diff. with 95% CI	Weight (%)
**Oesophageal baloon (transpulmonary pressure)**								
Wang 2020	30	17.54	52	28.33	21.37	50	1.67 [-5.90, 9.24]	4.53
Talmor 2008	18.3	13.8	30	14	11.6	31	4.30 [-2.09, 10.69]	6.23
Beitler 2019	11	8.27	102	9.5	6.77	98	1.50 [-0.60, 3.60]	35.99
							1.77 [-0.16, 3.69]	
**Respiratory static compliance**								
Pintado 2013	27.33	23.99	34	20.33	13.13	36	7.00 [-1.99, 15.99]	3.26
Cavalcanti 2017	18.2	22.4	501	19.2	25.9	509	-1.00 [-3.99, 1.99]	22.58
							1.83 [-5.67, 9.33]	
**Electrical impedance tomography**								
He 2021	15	13.67	61	10.6	5.94	56	4.40 [0.52, 8.28]	15.02
							4.40 [0.52, 8.28]	
**Lung ultrasound**								
Salem 2020	10	7.78	30	7.33	9.34	30	2.67 [-1.68, 7.02]	12.39
							2.67 [-1.68, 7.02]	
**Overall**							1.88 [0.22, 3.53]	

Heterogeneity: $\tau^2 = 0.84$, $I^2 = 16.58\%$, $H^2 = 1.20$

Test of $\theta_i = \theta_j$: $Q(6) = 7.19$, $p = 0.30$

Test of $\theta = 0$: $z = 2.22$, $p = 0.03$

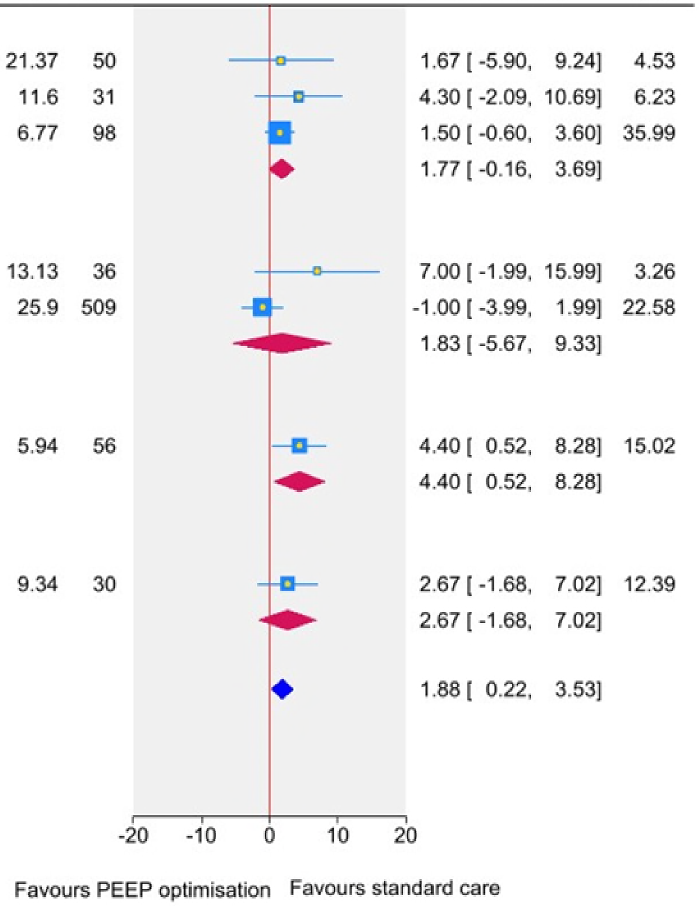

Random-effects DerSimonian–Laird model
Sorted by: _meta_weight

Figure S5: Forest plot- PEEP optimisation strategy v standard care; outcome: Hospital length of stay

	PEEP optimisation			Standard care				
Study ID	Mean	SD	Total	Mean	SD	Total	Mean diff. with 95% CI	Weight (%)
**Oesophageal baloon (transpulmonary pressure)**								
Wang 2020	32.33	19.82	52	30.33	17.56	50	2.00 [-5.28, 9.28]	18.82
Beitler 2019	17	12.78	102	15.67	12.04	98	1.33 [-2.11, 4.77]	20.17
							1.45 [-1.66, 4.57]	
**Respiratory static compliance**								
Cavalcanti 2017	25.5	32.3	501	26.2	31.7	509	-0.70 [-4.65, 3.25]	20.04
Pintado 2013	55	7	34	32	3	36	23.00 [20.50, 25.50]	20.37
							11.20 [-12.02, 34.43]	
**Intellivent-ASV**								
Yeremenko 2022	7.33	.77	40	8.67	1.54	40	-1.34 [-1.87, -0.81]	20.59
							-1.34 [-1.87, -0.81]	
**Overall**							4.91 [-5.83, 15.66]	

Heterogeneity: $\tau^2 = 145.83$, $I^2 = 98.86\%$, $H^2 = 87.48$

Test of $\theta_i = \theta_j$: Q(4) = 349.94, p = 0.00

Test of $\theta = 0$: z = 0.90, p = 0.37

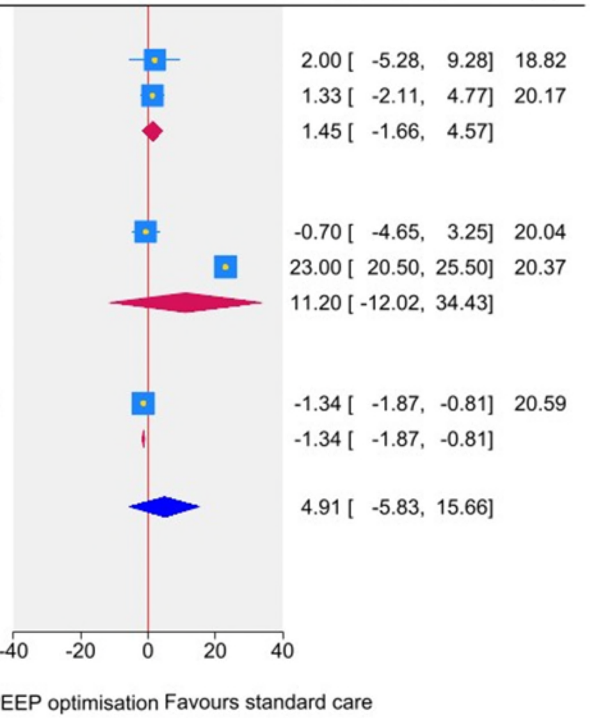

Random-effects DerSimonian–Laird model
Sorted by: _meta_weight

Figure S6: Forest plot- PEEP optimisation strategy v standard care; outcome: rate of pneumothorax

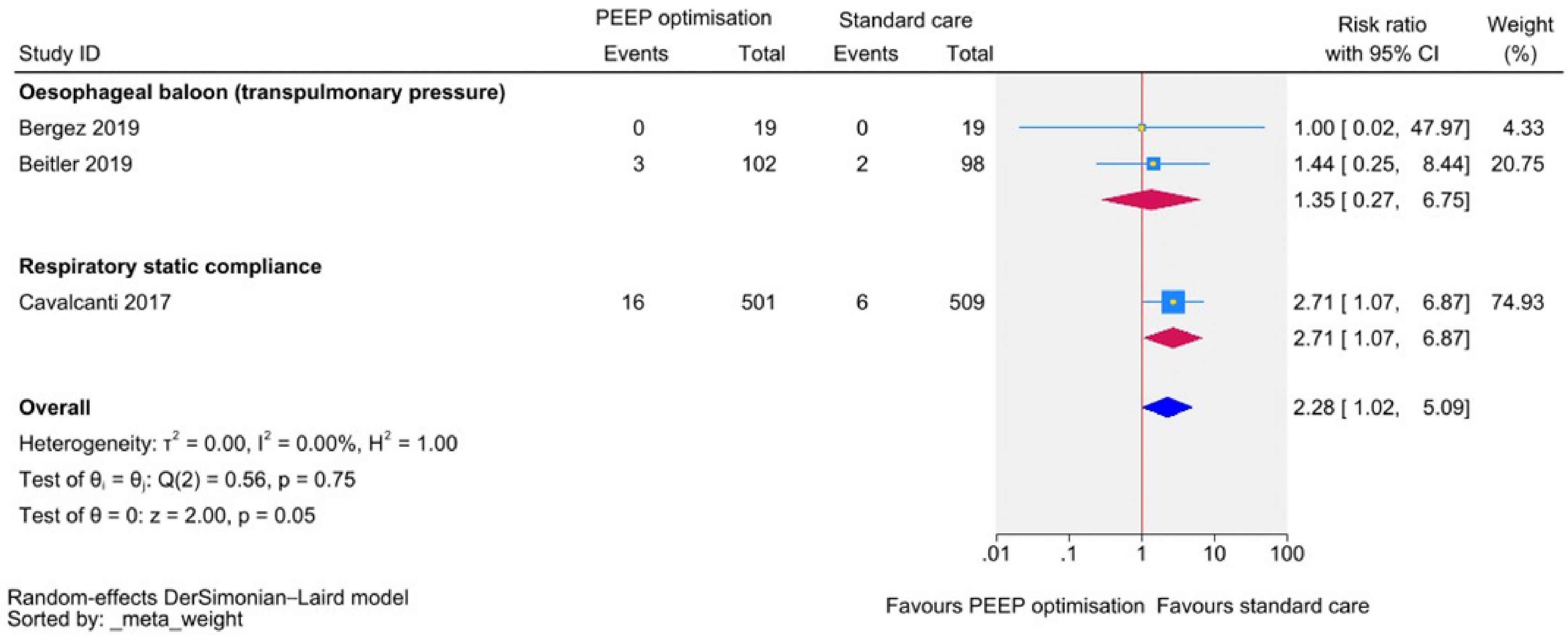

Figure S7: Forest plot- PEEP optimisation strategy v standard care; outcome: rate of barotrauma

Study ID	PEEP optimisation Events	PEEP optimisation Total	Standard care Events	Standard care Total	Risk ratio with 95% CI	Weight (%)
**Oesophageal baloon (transpulmonary pressure)**						
Wang 2020	3	52	5	50	0.58 [0.15, 2.29]	15.41
Beitler 2019	6	102	5	98	1.15 [0.36, 3.66]	19.25
					0.87 [0.36, 2.10]	
**Respiratory static compliance**						
Pintado 2013	6	34	6	36	1.06 [0.38, 2.97]	21.86
Cavalcanti 2017	28	501	8	509	3.56 [1.64, 7.73]	28.37
					2.04 [0.62, 6.66]	
**Electrical impedance tomography**						
Costa 2024	3	37	2	39	1.58 [0.28, 8.93]	11.10
					1.58 [0.28, 8.93]	
**Lung ultrasound**						
Salem 2020	0	30	1	30	0.33 [0.01, 7.87]	4.00
					0.33 [0.01, 7.87]	
**Overall**					1.38 [0.71, 2.67]	

Heterogeneity: $\tau^2 = 0.24$, $I^2 = 37.87\%$, $H^2 = 1.61$

Test of $\theta_i = \theta_j$: $Q(5) = 8.05$, $p = 0.15$

Test of $\theta = 0$: $z = 0.95$, $p = 0.34$

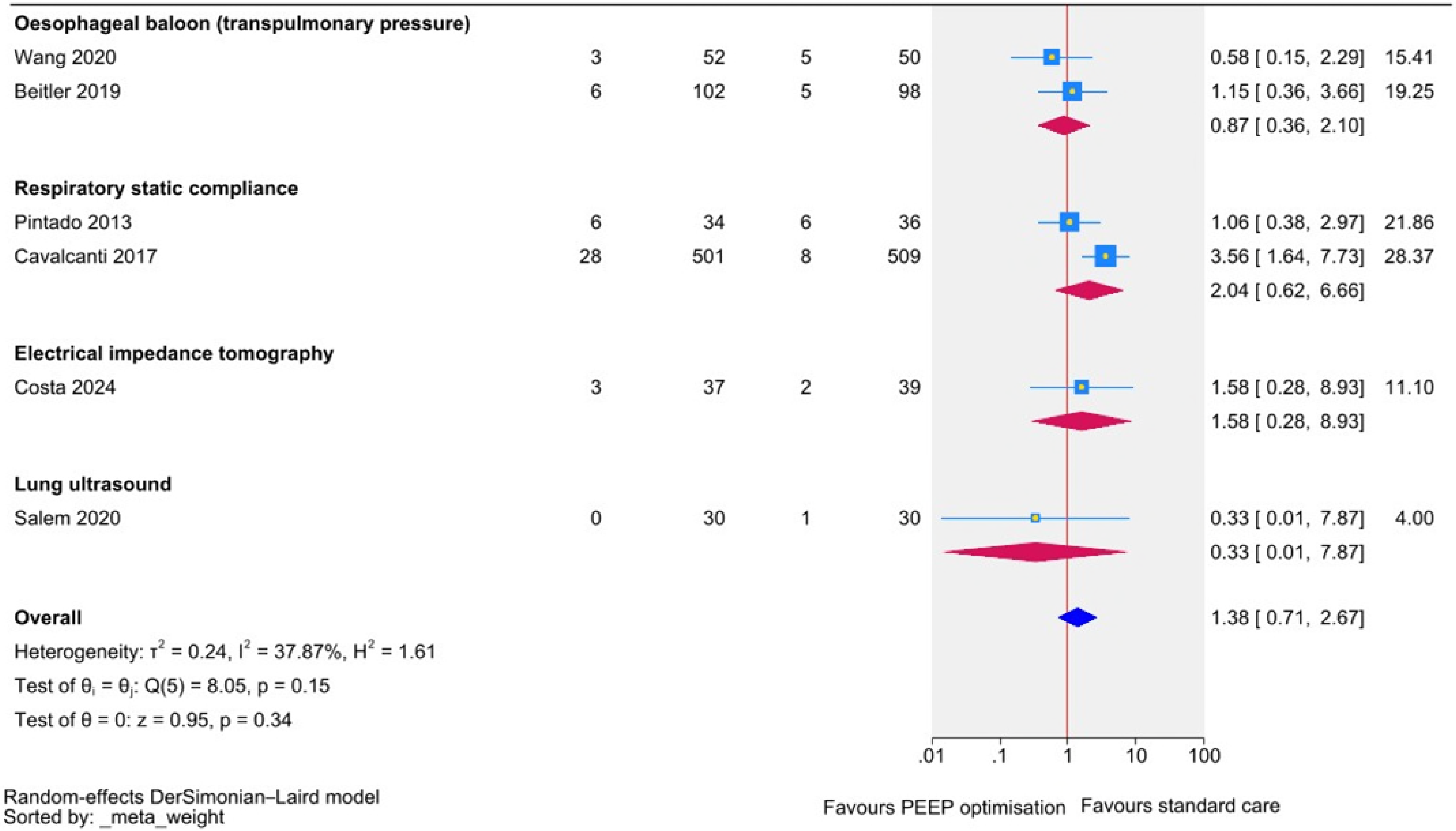

Random-effects DerSimonian–Laird model
Sorted by: _meta_weight

Figure S8: Forest plot- PEEP optimisation strategy v standard care; outcome: driving pressure

Study ID	PEEP optimisation Mean	PEEP optimisation SD	PEEP optimisation Total	Standard care Mean	Standard care SD	Standard care Total	Mean diff. with 95% CI	Weight (%)
**Oesophageal baloon (transpulmonary pressure)**								
Bergez 2019	14.2	5	19	16.4	7.8	19	-2.20 [-6.37, 1.97]	3.53
Wang 2020	9.6	2.2	52	12.6	2	50	-3.00 [-3.82, -2.18]	18.25
							-2.97 [-3.77, -2.17]	
**Intellivent-ASV**								
Buiteman-Kruizinga 2023	13.3	2.5	13	13.4	2.5	13	-0.10 [-2.02, 1.82]	10.39
Komnov 2023	7	1	16	9	2	16	-2.00 [-3.10, -0.90]	16.11
Yeremenko 2022	6.3	.8	40	7.8	1.5	40	-1.50 [-2.03, -0.97]	20.22
							-1.48 [-2.16, -0.81]	
**Electrical impedance tomography**								
Jimenez 2023	10.6	1.82	12	11.91	1.57	12	-1.31 [-2.67, 0.05]	14.09
He 2021	13	3.04	61	12.67	3.8	56	0.33 [-0.91, 1.57]	14.97
							-0.47 [-2.07, 1.14]	
**Nitrogen multiple breath washout/wash-in**								
Rollas 2022	15.33	4.24	11	14.67	7.63	11	0.66 [-4.50, 5.82]	2.44
							0.66 [-4.50, 5.82]	
**Overall**							-1.38 [-2.23, -0.53]	

Heterogeneity: $\tau^2 = 0.87$, $I^2 = 71.45\%$, $H^2 = 3.50$

Test of $\theta_i = \theta_j$: $Q(7) = 24.52$, $p = 0.00$

Test of $\theta = 0$: $z = -3.17$, $p = 0.00$

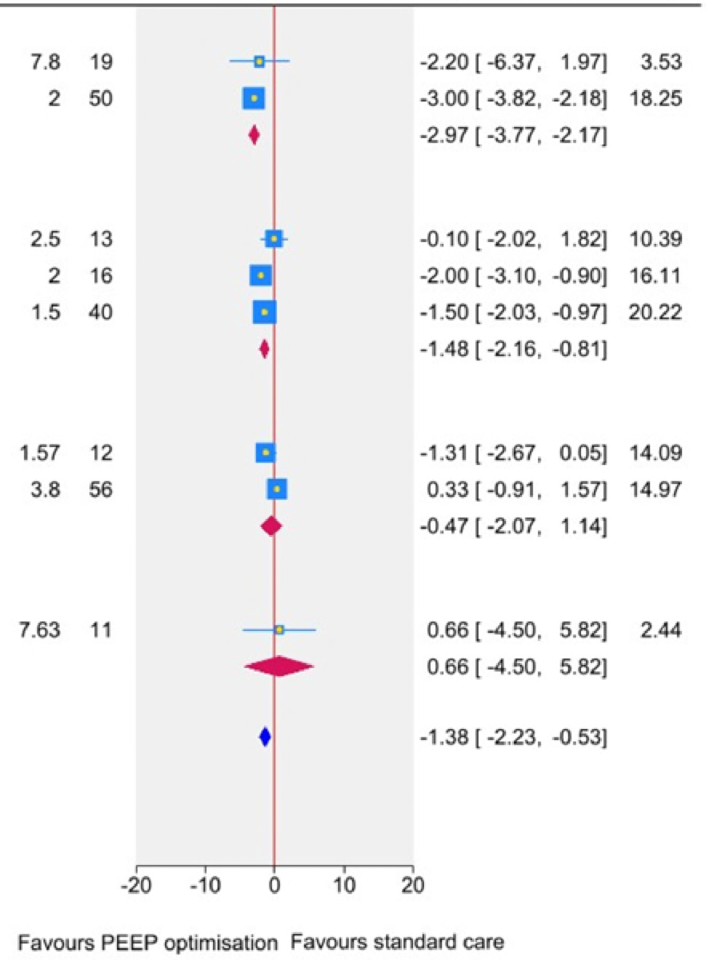

Random-effects DerSimonian–Laird model
Sorted by: _meta_weight

Figure S9: Forest plot- PEEP optimisation strategy v standard care; outcome: mechanical power

Study ID	PEEP optimisation Mean	PEEP optimisation SD	PEEP optimisation Total	Standard care Mean	Standard care SD	Standard care Total	Mean diff. with 95% CI	Weight (%)
**Intellivent-ASV**								
Komnov 2023	10	3	16	15	5	16	-5.00 [-7.86, -2.14]	5.33
Buiteman-Kruizinga 2023	17.2	2.6	13	17.6	3.1	13	-0.40 [-2.60, 1.80]	8.49
Yeremenko 2022	7.7	2.3	40	9	3.1	40	-1.30 [-2.50, -0.10]	21.58
							-1.98 [-4.14, 0.18]	
**Nitrogen multiple breath washout/wash-in**								
Di 2020	15.22	1.25	41	17.03	1.5	37	-1.81 [-2.42, -1.20]	41.41
							-1.81 [-2.42, -1.20]	
**Electrical impedance tomography**								
Jimenez 2023	21.77	6.53	12	23.52	4.5	12	-1.75 [-6.24, 2.74]	2.28
							-1.75 [-6.24, 2.74]	
**Oesophageal baloon (transpulmonary pressure)**								
Wang 2020	6.4	3.3	52	8.2	3	50	-1.80 [-3.03, -0.57]	20.91
							-1.80 [-3.03, -0.57]	
**Overall**							-1.75 [-2.44, -1.06]	

Heterogeneity: $\tau^2 = 0.20$, $I^2 = 28.62\%$, $H^2 = 1.40$

Test of $\theta_i = \theta_j$: $Q(5) = 7.01$, $p = 0.22$

Test of $\theta = 0$: $z = -4.96$, $p = 0.00$

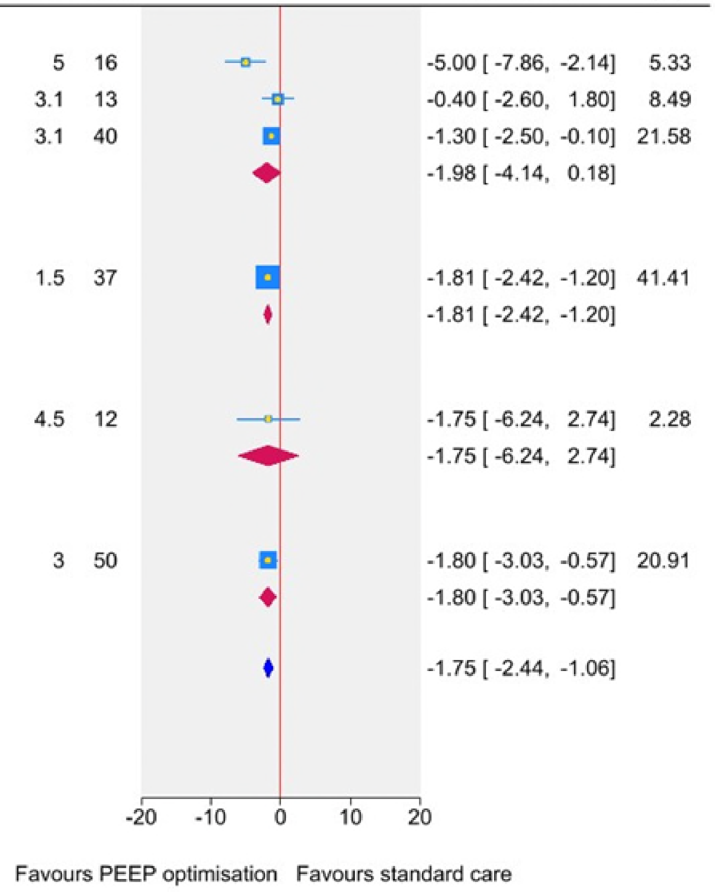

Random-effects DerSimonian–Laird model
Sorted by: _meta_weight

Figure S10: Forest plot- PEEP optimisation strategy v standard care; outcome: Plateau pressure

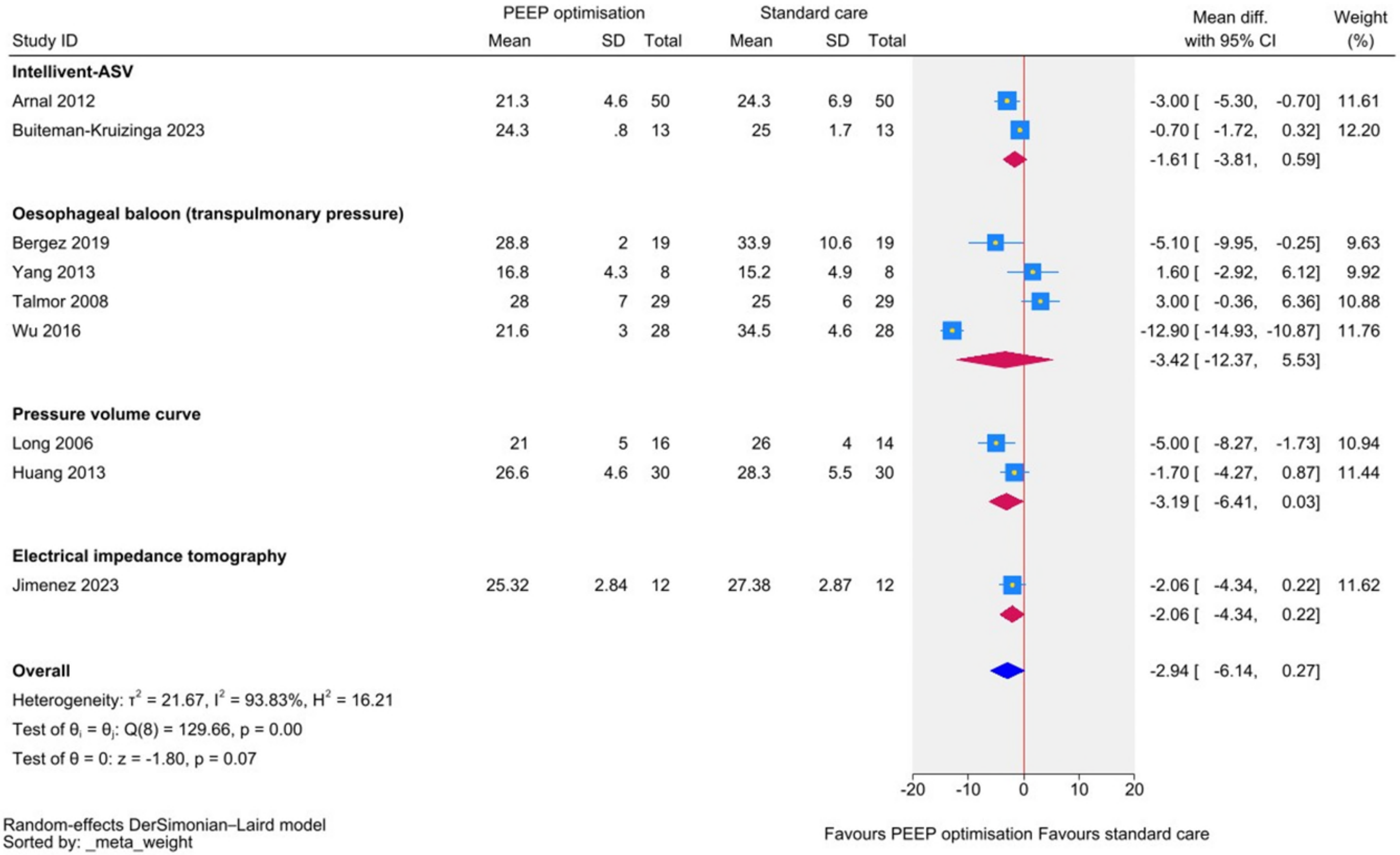

Figure S11: Forest plot- PEEP optimisation strategy v standard care; outcome: Sensitivity analysis by physiological parameter grouping: Duration of mechanical ventilation

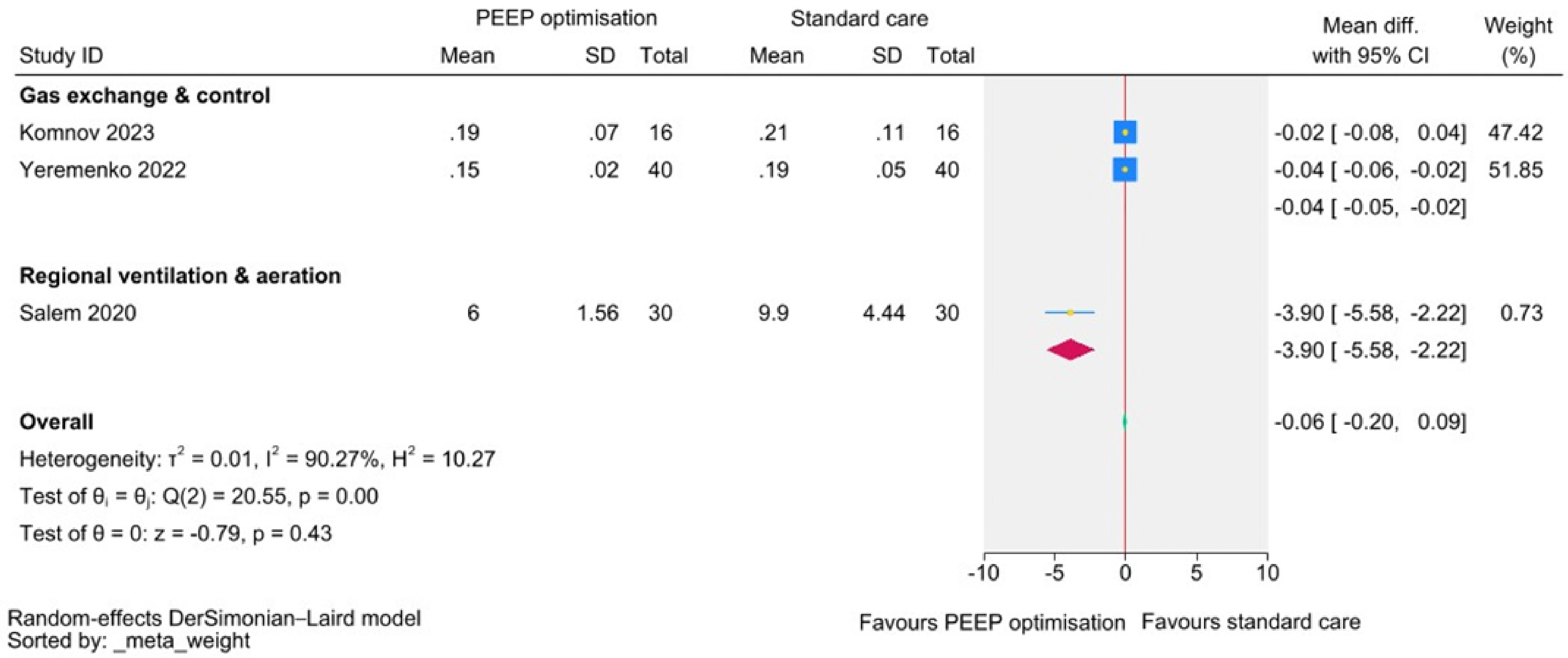

Figure S12: Forest plot- PEEP optimisation strategy v standard care; outcome: Sensitivity analysis by physiological parameter grouping: 28-day mortality

Study ID	PEEP optimisation Events	PEEP optimisation Total	Standard care Events	Standard care Total	Risk ratio with 95% CI	Weight (%)
**Respiratory mechanics**						
Talmor 2008	5	30	12	31	0.43 [0.17, 1.07]	6.73
Long 2006	6	16	8	14	0.66 [0.30, 1.43]	8.19
Pintado 2013	7	34	14	36	0.53 [0.24, 1.15]	8.21
Amato 1998	11	29	17	24	0.54 [0.31, 0.91]	11.79
Nguyen 2019	10	20	14	20	0.71 [0.42, 1.21]	11.92
Beitler 2019	33	102	30	98	1.06 [0.70, 1.59]	13.97
Cavalcanti 2017	277	501	251	509	1.12 [1.00, 1.26]	18.56
					0.76 [0.56, 1.04]	
**Regional ventilation & aeration**						
Salem 2020	2	30	9	30	0.22 [0.05, 0.94]	3.41
He 2021	13	61	15	56	0.80 [0.42, 1.52]	9.93
					0.50 [0.15, 1.66]	
**Lung volume**						
Di 2020	6	41	13	37	0.42 [0.18, 0.98]	7.28
					0.42 [0.18, 0.98]	
**Overall**					0.69 [0.52, 0.93]	

Heterogeneity: $\tau^2 = 0.12$, $I^2 = 65.57\%$, $H^2 = 2.90$

Test of $\theta_i = \theta_j$: Q(9) = 26.14, p = 0.00

Test of $\theta = 0$: z = -2.43, p = 0.01

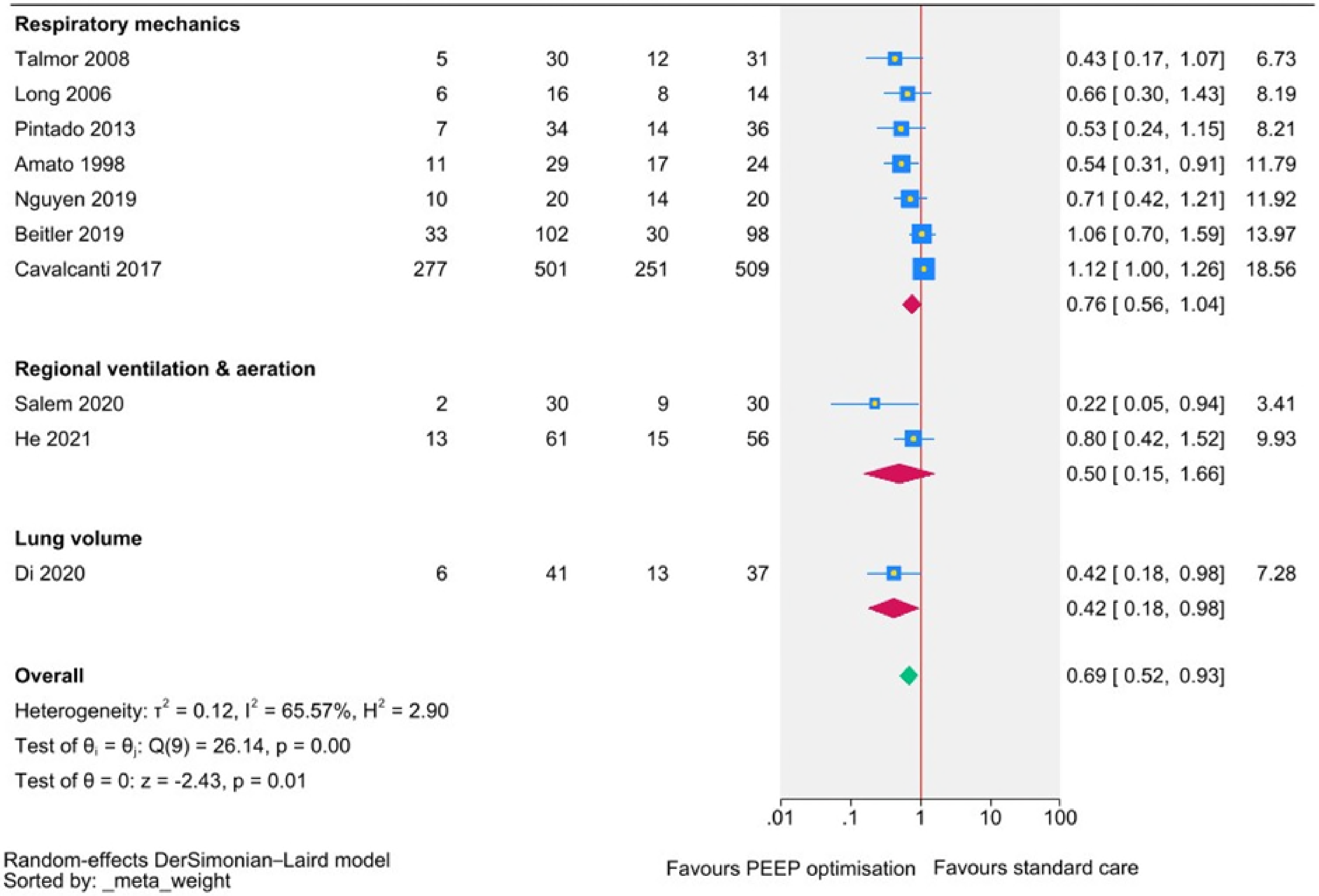

Random-effects DerSimonian–Laird model
Sorted by: _meta_weight

Figure S13: Funnel plot - PEEP optimisation strategy v standard care; outcome: 28 day mortality

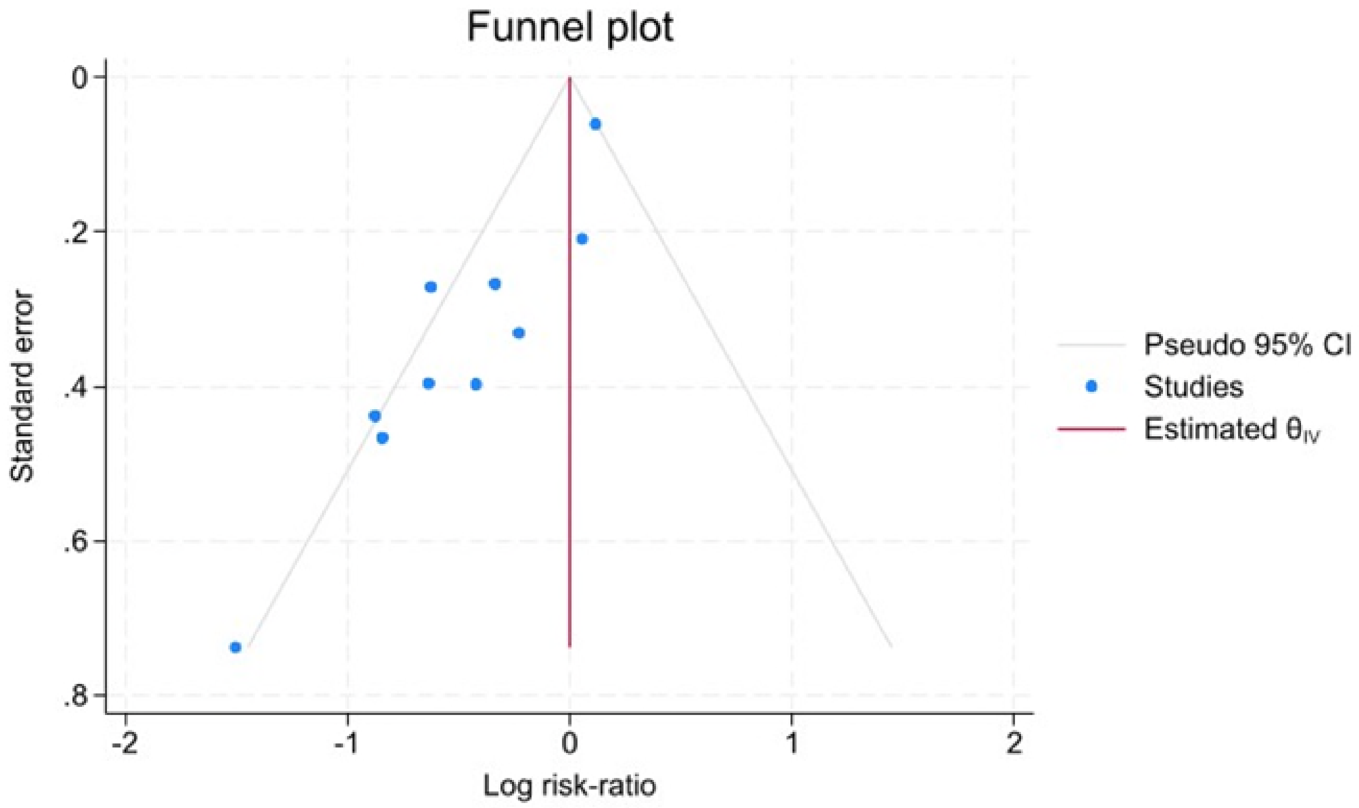

GRADE tables

Oesophageal balloon (transpulmonary pressure) compared to standard care											
**Certainty assessment**							**Summary of findings**				
							**Study event rates (%)**			**Anticipated absolute effects**	
**Participants (studies) Follow-up**	**Risk of bias**	**Inconsistency**	**Indirectness**	**Imprecision**	**Publication bias**	**Overall certainty of evidence**	**With standard care**	**With Oesophageal balloon (transpulmonary pressure)**	**Relative effect (95% CI)**	**Risk with standard care**	**Risk difference with Oesophageal balloon (transpulmonary pressure)**
**28-day mortality**											
325 (3 RCTs)	serious	serious[a]	not serious	extremely serious[b]	none	⨁◯◯◯ Very low[a,b]	48/150 (32.0%)	43/175 (24.6%)	**RR 0.65** (0.32 to 1.31)	48/150 (32.0%)	**112 fewer per 1,000** (from 218 fewer to 99 more)
**ICU length of stay**											
325 (3 RCTs)	serious[c]	not serious	not serious	serious[d]	none	⨁⨁◯◯ Low[c,d]	150	175	-	150	MD 0.68 lower (2.8 lower to 1.43 higher)
**Ventilator free days**											
261 (2 RCTs)	not serious	not serious	not serious	very serious[d]	none	⨁⨁◯◯ Low[d]	129	132	-	129	MD 0.3 higher (3.71 lower to 4.3 higher)
**Duration of mechanical ventilation**											
64 (1 RCT)	serious[c]	not serious	not serious	very serious[e]	none	⨁◯◯◯ Very low[c,e]	21	43	-	21	MD 1.47 lower (1.95 lower to 0.99 lower)
**Pneumothorax**											

**Oesophageal balloon (transpulmonary pressure) compared to standard care**											
**Certainty assessment**							**Summary of findings**				
238 (2 RCTs)	serious[c]	not serious	not serious	extremely serious[d]	none	⊕◯◯◯ Very low[c,d]	2/117 (1.7%)	3/121 (2.5%)	**RR 1.35** (0.27 to 6.75)	2/117 (1.7%)	**6 more per 1,000** (from 12 fewer to 98 more)
**Barotrauma**											
302 (2 RCTs)	not serious	not serious	not serious	extremely serious[b]	none	⊕◯◯◯ Very low[b]	10/148 (6.8%)	9/154 (5.8%)	**RR 0.87** (0.36 to 2.10)	10/148 (6.8%)	**9 fewer per 1,000** (from 43 fewer to 74 more)
**Renal replacement therapy**											
196 (1 RCT)	not serious	not serious	not serious	extremely serious[e]	none	⊕◯◯◯ Very low[e]	32/96 (33.3%)	21/100 (21.0%)	**RR 0.63** (0.39 to 1.01)	32/96 (33.3%)	**123 fewer per 1,000** (from 203 fewer to 3 more)
**Driving pressure**											
140 (2 RCTs)	serious[c]	serious[a]	not serious	not serious	none	⊕⊕◯◯ Low[a,c]	69	71	-	69	MD 2.97 lower (3.77 lower to 2.17 lower)
**PEEP**											
319 (6 RCTs)	serious[f]	serious[a]	not serious	very serious[g]	none	⊕◯◯◯ Very low[a,f,g]	147	172	-	147	MD 2.52 higher (3.06 lower to 8.1 higher)
**Mechanical power**											
102 (1 RCT)	not serious	not serious	not serious	extremely serious[e]	none	⊕◯◯◯ Very low[e]	50	52	-	50	MD 1.8 lower (3.03 lower to 0.57 lower)

Oesophageal balloon (transpulmonary pressure) compared to standard care											
Certainty assessment							Summary of findings				
**Plateau pressure**											
232 (5 RCTs)	serious[h]	serious[a]	not serious	extremely serious[g]	none	⨁◯◯◯ Very low[a,g,h]	105	127	-	105	MD 0.08 higher (12.08 lower to 12.23 higher)
**Hospital length of stay**											
302 (2 RCTs)	not serious	not serious	not serious	extremely serious[d]	none	⨁◯◯◯ Very low[d]	148	154	-	148	MD 1.45 higher (1.66 lower to 4.57 higher)

CI: confidence interval; **MD:** mean difference; **RR:** risk ratio

Explanations

a. Siginificant heterogeneity was found

b. Very wide confidence interval suggesting appreciable benefit and appreciable harm.

c. One study was rated to be at high risk of bias

d. Wide confidence interval suggesting appreciable benefit and appreciable harm

e. Single study with a small sample size

f. One study was rated to be at high risk of bias and three had some concerns

g. Very wide confidence interval

h. Two studies rated to be at high risk of bias and two were deemed as having some concerns

Low certainty of evidence implies that the available evidence is limited and the true effect may be substantially different from the estimate.

Very low certainty of evidence indicates that the available evidence is insufficient to support any firm conclusions.

Electrical impedance tomography compared to standard care

Certainty assessment							Summary of findings				
Participants (studies) Follow-up	Risk of bias	Inconsistency	Indirectness	Imprecision	Publication bias	Overall certainty of evidence	Study event rates (%) With standard care	Study event rates (%) With electrical impedance tomography	Relative effect (95% CI)	Anticipated absolute effects: Risk with standard care	Anticipated absolute effects: Risk difference with electrical impedance tomography
**28-day mortality**											
117 (1 RCT)	serious[a]	not serious	not serious	extremely serious[b]	none	⨁◯◯◯ Very low[a,b]	15/56 (26.8%)	13/61 (21.3%)	**RR 0.80** (0.42 to 1.52)	15/56 (26.8%)	**54 fewer per 1,000** (from 155 fewer to 139 more)
**Ventilator-free days**											
117 (1 RCT)	serious[a]	not serious	not serious	extremely serious[b]	none	⨁◯◯◯ Very low[a,b]	56	61	-	56	MD 1.84 lower (8.31 lower to 4.63 higher)
**ICU length of stay**											
117 (1 RCT)	serious[a]	not serious	not serious	very serious[c]	none	⨁◯◯◯ Very low[a,c]	56	61	-	56	MD 4.4 higher (0.52 higher to 8.28 higher)
**Barotrauma**											
76 (1 RCT)	not serious	not serious	not serious	extremely serious[b]	none	⨁◯◯◯ Very low[b]	2/39 (5.1%)	3/37 (8.1%)	**RR 1.58** (0.28 to 8.93)	2/39 (5.1%)	**30 more per 1,000** (from 37 fewer to 407 more)
**Renal replacement therapy**											

Electrical impedance tomography compared to standard care											
**Certainty assessment**							**Summary of findings**				
76 (1 RCT)	not serious	not serious	not serious	extremely serious[b]	none	⊕◯◯◯ Very low[b]	13/39 (33.3%)	14/37 (37.8%)	**RR 1.14** (0.62 to 2.08)	13/39 (33.3%)	**47 more per 1,000** (from 127 fewer to 360 more)
**Shock**											
76 (1 RCT)	not serious	not serious	not serious	extremely serious[b]	none	⊕◯◯◯ Very low[b]	25/39 (64.1%)	25/37 (67.6%)	**RR 1.05** (0.76 to 1.46)	25/39 (64.1%)	**32 more per 1,000** (from 154 fewer to 295 more)
**Driving pressure**											
141 (2 RCTs)	serious[d]	serious[e]	not serious	serious[f]	none	⊕◯◯◯ Very low[d,e,f]	68	73	-	68	MD 0.47 lower (2.07 lower to 1.14 higher)
**PEEP**											
223 (4 RCTs)	serious[g]	serious[e]	not serious	very serious[f]	none	⊕◯◯◯ Very low[e,f,g]	109	114	-	109	MD 0.75 lower (3.66 lower to 2.17 higher)
**Mechanical power**											
24 (1 RCT)	serious[a]	not serious	not serious	extremely serious[f]	none	⊕◯◯◯ Very low[a,f]	12	12	-	12	MD 1.75 lower (6.24 lower to 2.74 higher)
**Plateau pressure**											
24 (1 RCT)	serious[a]	not serious	not serious	very serious[f]	none	⊕◯◯◯ Very low[a,f]	12	12	-	12	MD 2.06 lower (4.34 lower to 0.22 higher)

CI: confidence interval; **MD:** mean difference; **RR:** risk ratio

Explanations

a. The study was deemed as having some concerns

b. Very wide confidence interval that includes appreciable benefit and appreciable harm

c. Single study with a small sample size

d. Two studies were deemed as having some concerns

e. Significant heterogeneity was found

f. Wide confidence interval

g. One study was rated to be at high risk of bias and three studies were deemed as having some concerns

Low certainty of evidence implies that the available evidence is limited and the true effect may be substantially different from the estimate.

Very low certainty of evidence indicates that the available evidence is insufficient to support any firm conclusions.

Pressure volume curve compared to standard care											
**Certainty assessment**							**Summary of findings**				
**Participants (studies) Follow-up**	**Risk of bias**	**Inconsistency**	**Indirectness**	**Imprecision**	**Publication bias**	**Overall certainty of evidence**	**Study event rates (%)**		**Relative effect (95% CI)**	**Anticipated absolute effects**	
							**With standard care**	**With Pressure volume curve**		**Risk with standard care**	**Risk difference with Pressure volume curve**
**28-day mortality**											
123 (3 RCTs)	serious[a]	not serious	not serious	serious[b]	none	⨁⨁◯◯ Low[a,b]	39/58 (67.2%)	27/65 (41.5%)	**RR 0.63** (0.45 to 0.88)	39/58 (67.2%)	**249 fewer per 1,000** (from 370 fewer to 81 fewer)
**PEEP**											
30 (1 RCT)	serious[c]	not serious	not serious	very serious[d]	none	⨁◯◯◯ Very low[c,d]	14	16	-	14	MD 5 lower (6.8 lower to 3.2 lower)
**Plateau pressure**											
90 (2 RCTs)	serious[e]	not serious	not serious	very serious[f]	none	⨁◯◯◯ Very low[e,f]	44	46	-	44	MD 3.19 lower (6.41 lower to 0.03 higher)

CI: confidence interval; **MD:** mean difference; **RR:** risk ratio

Explanations

a. One study was rated to be at high risk of bias and we had some concerns for another.

b. Optimal information size was not reached

c. The study was deemed as having some concerns

d. Single study with small sample size

e. Two studies deemed as having some concerns

f. Wide confidence interval

Low certainty of evidence implies that the available evidence is limited and the true effect may be substantially different from the estimate.

Very low certainty of evidence indicates that the available evidence is insufficient to support any firm conclusions.

Fully automated closed loop ventilation (Intellivent-ASV) compared to standard care											
**Certainty assessment**							**Summary of findings**				
**Participants (studies) Follow-up**	**Risk of bias**	**Inconsistency**	**Indirectness**	**Imprecision**	**Publication bias**	**Overall certainty of evidence**	**Study event rates (%)**		**Relative effect (95% CI)**	**Anticipated absolute effects**	
							**With standard care**	**With Intellivent-ASV**		**Risk with standard care**	**Risk difference with Intellivent-ASV**
**Duration of respiratory support in the ICU**											
112 (2 RCTs)	serious[a]	serious[b]	not serious	serious[c]	none	⨁◯◯◯ Very low[a,b,c]	56	56	-	56	MD **0.1 days lower** (1.57 lower to 1.36 higher)
**Hospital length of stay**											
80 (1 RCT)	serious[d]	not serious	not serious	very serious[e]	none	⨁◯◯◯ Very low[d,e]	40	40	-	40	MD **1.34 lower** (1.87 lower to 0.81 lower)
**Driving pressure**											
138 (3 RCTs)	serious[f]	not serious	not serious	serious[g]	none	⨁⨁◯◯ Low[f,g]	69	69	-	69	MD **1.48 lower** (2.16 lower to 0.81 lower)
**PEEP**											
768 (5 RCTs)	serious[h]	serious[b]	not serious	serious[c]	none	⨁◯◯◯ Very low[b,c,h]	384	384	-	384	MD **1.02 lower** (2.05 lower to 0)
**Mechanical power**											
138 (3 RCTs)	serious[f]	serious[b]	not serious	serious[c]	none	⨁◯◯◯ Very low[b,c,f]	69	69	-	69	MD **1.98 lower** (4.14 lower to 0.18 higher)

Fully automated closed loop ventilation (Intellivent-ASV) compared to standard care											
**Certainty assessment**							**Summary of findings**				
**Plateau pressure**											
126 (2 RCTs)	serious[i]	serious[b]	not serious	serious[c]	none	⊕◯◯◯ Very low[b,c,i]	63	63	-	63	MD **1.61 lower** (3.81 lower to 0.59 higher)

CI: confidence interval; **MD:** mean difference

Explanations

a. One study was rated to be at high risk of bias and we had some concerns for the other.

b. Significant heterogeneity was found.

c. Wide confidence interval

d. The study was deemed as having some concerns

e. Single study with small sample size

f. One study was rated to be at high risk of bias and two studies were deemed as having some concerns

g. Three trials with small sample sizes

h. One study was rated to be at high risk of bias and four studies were deemed as having some concerns

i. Two studies deemed as having some concerns

Low certainty of evidence implies that the available evidence is limited and the true effect may be substantially different from the estimate.

Very low certainty of evidence indicates that the available evidence is insufficient to support any firm conclusions.

Static respiratory compliance measurement compared to standard care											
**Certainty assessment**							**Summary of findings**				
							**Study event rates (%)**			**Anticipated absolute effects**	
**Participants (studies) Follow-up**	**Risk of bias**	**Inconsistency**	**Indirectness**	**Imprecision**	**Publication bias**	**Overall certainty of evidence**	**With standard care**	**With Intellivent-ASV**	**Relative effect (95% CI)**	**Risk with standard care**	**Risk difference with Intellivent-ASV**
**28-day mortality**											
1080 (2 RCTs)	not serious	serious[a]	not serious	extremely serious[b]	none	⨁◯◯◯ Very low[a,b]	265/545 (48.6%)	284/535 (53.1%)	**RR 0.85** (0.42 to 1.73)	265/545 (48.6%)	**73 fewer per 1,000** (from 282 fewer to 355 more)
**Barotrauma**											
1080 (2 RCTs)	not serious	serious[a]	not serious	extremely serious[b]	none	⨁◯◯◯ Very low[a,b]	14/545 (2.6%)	34/535 (6.4%)	**RR 2.04** (0.62 to 6.66)	14/545 (2.6%)	**27 more per 1,000** (from 10 fewer to 145 more)
**Hospital length of stay**											
1080 (2 RCTs)	not serious	serious[a]	not serious	extremely serious[b]	none	⨁◯◯◯ Very low[a,b]	545	535	-	545	MD 11.2 higher (12.02 lower to 34.43 higher)
**ICU length of stay**											
1080 (2 RCTs)	not serious	serious[a]	not serious	very serious[b]	none	⨁◯◯◯ Very low[a,b]	545	535	-	545	MD 1.83 higher (5.67 lower to 9.33 higher)
**Ventilator free days**											

**Static respiratory compliance measurement compared to standard care**											
**Certainty assessment**							**Summary of findings**				
1080 (2 RCTs)	not serious	not serious	not serious	serious[c]	none	⊕⊕⊕◯ Moderate[c]	545	535	-	545	MD 1.04 lower (2.05 lower to 0.03 lower)
**Pneumothorax**											
1010 (1 RCT)	not serious	not serious	not serious	very serious[d]	none	⊕⊕◯◯ Low[d]	6/509 (1.2%)	16/501 (3.2%)	**RR 2.28** (1.02 to 5.09)	6/509 (1.2%)	**15 more per 1,000** (from 0 fewer to 48 more)

CI: confidence interval; **MD:** mean difference; **RR:** risk ratio

Explanations

a. Significant heterogeneity was found

b. Very wide confidence interval suggesting appreciable benefit and appreciable harm

c. Only two trials contributed to the meta-analysis and overall sample size is quite small.

d. Wide confidence interval spanning two thresholds

Moderate certainty of evidence suggests that the available evidence is sufficient to support a conclusion, but further research may still impact the confidence in the estimate.

Low certainty of evidence implies that the available evidence is limited and the true effect may be substantially different from the estimate.

Very low certainty of evidence indicates that the available evidence is insufficient to support any firm conclusions.

Nitrogen wash-in/washout compared to standard care												
**Certainty assessment**							**Summary of findings**					
**Participants (studies) Follow-up**	**Risk of bias**	**Inconsistency**	**Indirectness**	**Imprecision**	**Publication bias**	**Overall certainty of evidence**	**Study event rates (%)**		**Relative effect (95% CI)**	**Anticipated absolute effects**	
							**With standard care**	**With nitrogen wash-in/washout**		**Risk with standard care**	**Risk difference with nitrogen wash-in/washout**
**28-day mortality**											
78 (1 RCT)	serious[a]	not serious	not serious	very serious[b]	none	⊕◯◯◯ Very low[a,b]	13/37 (35.1%)	6/41 (14.6%)	**RR 0.42** (0.18 to 0.98)	13/37 (35.1%)	**204 fewer per 1,000** (from 288 fewer to 7 fewer)
**Driving pressure**											
22 (1 RCT)	serious[c]	not serious	not serious	very serious[b]	none	⊕◯◯◯ Very low[b,c]	11	11	-	11	MD 0.66 higher (4.5 lower to 5.82 higher)
**PEEP**											
100 (2 RCTs)	serious[d]	not serious	not serious	serious[e]	none	⊕⊕◯◯ Low[d,e]	48	52	-	48	MD 2.1 higher (1.5 higher to 2.7 higher)
**Mechanical power**											
78 (1 RCT)	serious[c]	not serious	not serious	very serious[e]	none	⊕◯◯◯ Very low[c,e]	37	41	-	37	MD 1.81 lower (2.42 lower to 1.2 lower)

CI: confidence interval; **MD:** mean difference; **RR:** risk ratio

Explanations

a. The study was rated to be at high risk of bias

b. Single study and a very wide confidence interval

c. The study was rated as having some concerns

d. Two studies deemed as having some concerns

e. Small sample size

Low certainty of evidence implies that the available evidence is limited and the true effect may be substantially different from the estimate.

Very low certainty of evidence indicates that the available evidence is insufficient to support any firm conclusions.

Lung ultrasound compared to standard care											
**Certainty assessment**							**Summary of findings**				
**Participants (studies) Follow-up**	**Risk of bias**	**Inconsistency**	**Indirectness**	**Imprecision**	**Publication bias**	**Overall certainty of evidence**	**Study event rates (%)**		**Relative effect (95% CI)**	**Anticipated absolute effects**	
							**With standard care**	**With lung ultrasound**		**Risk with standard care**	**Risk difference with lung ultrasound**
**28-day mortality**											
60 (1 RCT)	serious[a]	not serious	not serious	very serious[b]	none	⨁◯◯◯ Very low[a,b]	9/30 (30.0%)	2/30 (6.7%)	**RR 0.22** (0.05 to 0.94)	9/30 (30.0%)	**234 fewer per 1,000** (from 285 fewer to 18 fewer)
**Duration of mechanical ventilation**											
60 (1 RCT)	serious[a]	not serious	not serious	very serious[b]	none	⨁◯◯◯ Very low[a,b]	30	30	-	30	MD 3.9 lower (5.58 lower to 2.22 lower)
**Ventilator free days**											
60 (1 RCT)	serious[a]	not serious	not serious	very serious[b]	none	⨁◯◯◯ Very low[a,b]	30	30	-	30	MD 9 higher (2.84 higher to 15.16 higher)
**ICU length of stay**											
60 (1 RCT)	serious[a]	not serious	not serious	very serious[b]	none	⨁◯◯◯ Very low[a,b]	30	30	-	30	MD 2.67 higher (1.68 lower to 7.02 higher)
**Barotrauma**											
60 (1 RCT)	serious[a]	not serious	not serious	extremely serious[c]	none	⨁◯◯◯ Very low[a,c]	1/30 (3.3%)	0/30 (0.0%)	**RR 0.33** (0.01 to 7.87)	1/30 (3.3%)	**22 fewer per 1,000** (from 33 fewer to 229 more)

Lung ultrasound compared to standard care											
Certainty assessment							Summary of findings				
**PEEP**											
40 (1 RCT)	serious[a]	not serious	not serious	very serious[b]	none	⨁◯◯◯ Very low[a,b]	20	20	-	20	MD 3 higher (0.81 higher to 5.19 higher)

CI: confidence interval; **MD:** mean difference; **RR:** risk ratio

Explanations

a. The study was deemed as having some concerns

b. Single study and a small sample size

c. Very wide confidence interval suggesting appreciable benefit and appreciable harm

Very low certainty of evidence indicates that the available evidence is insufficient to support any firm conclusions.
